# Supplementary material for: Impact of COVID-19 on household food insecurity and interlinkages with child feeding practices and coping strategies in Uttar Pradesh, India: a longitudinal community-based study
Source: BMJ Open. 2021 Apr 21;11(4):e048738. doi: 10.1136/bmjopen-2021-048738 (PMC8061560; doi:10.1136/bmjopen-2021-048738)
Supplement: Supplementary data [file bmjopen-2021-048738supp001.pdf]

Supplemental Table 1: Food items from the maternal recall in the 24 hours prior to the survey

No	Food items
1.	Porridge, bread, rice, noodles, or other foods made from grains
2.	Pumpkin, carrots, squash, or sweet potatoes that are yellow or orange inside
3.	White potatoes, white yams, manioc, cassava, or any other foods made from roots
4.	Any dark green leafy vegetables
5.	Ripe mangoes, ripe papayas, or other local vitamin A-rich fruits
6.	Any other fruits or vegetables
7.	Liver, kidney, heart, or other organ meats
8.	Any meat, such as beef, pork, lamb, goat, chicken, or duck
9.	Eggs
10.	Fresh or dried fish, shellfish, or seafood
11.	Any foods made from beans, peas, lentils, nuts, or seeds
12.	Cheese, yogurt, or other milk products
13.	Any oil, fats, or butter, or foods made with any of these
14.	Any sugary foods such as chocolates, sweets, candies, pastries, cakes, or biscuits
15.	Condiments for flavor, such as chilies, spices, herbs, or fish powder
16.	Grubs, snails, or insects
17.	Foods made with red palm oil, red palm nut, or red palm nut pulp sauce
18.	Baby formula
