# Supplementary material for: Impact of COVID-19 on household food insecurity and interlinkages with child feeding practices and coping strategies in Uttar Pradesh, India: a longitudinal community-based study
Source: BMJ Open. 2021 Apr 21;11(4):e048738. doi: 10.1136/bmjopen-2021-048738 (PMC8061560; doi:10.1136/bmjopen-2021-048738)
Supplement: Author's manuscript [file bmjopen-2021-048738.draft_revisions.pdf]

BMJ Open is committed to open peer review. As part of this commitment we make the peer review history of every article we publish publicly available.

When an article is published we post the peer reviewers' comments and the authors' responses online. We also post the versions of the paper that were used during peer review. These are the versions that the peer review comments apply to.

The versions of the paper that follow are the versions that were submitted during the peer review process. They are not the versions of record or the final published versions. They should not be cited or distributed as the published version of this manuscript.

BMJ Open is an open access journal and the full, final, typeset and author-corrected version of record of the manuscript is available on our site with no access controls, subscription charges or pay-per-view fees (http://bmjopen.bmj.com).

If you have any questions on BMJ Open's open peer review process please email info.bmjopen@bmj.com

# The impact of COVID-19 on household food insecurity and interlinkages with child feeding practices and coping strategies in Uttar Pradesh, India

| | |
|---|---|
| Journal: | *BMJ Open* |
| Manuscript ID | bmjopen-2021-048738 |
| Article Type: | Original research |
| Date Submitted by the Author: | 06-Jan-2021 |
| Complete List of Authors: | Nguyen, Phuong Hong; International Food Policy Research Institute, Poverty, Health and Nutrition Division<br>Kachwaha, Shivani; International Food Policy Research Institute, Poverty, Health and Nutrition Division<br>Pant, Anjali; International Food Policy Research Institute, Poverty, Health and Nutrition Division<br>Tran, Lan; FHI Solutions, Alive &Thrive<br>Ghosh, Sebanti; FHI Solutions, Alive &Thrive<br>Sharma , Praveen ; FHI Solutions, Alive &Thrive<br>Shastri , Vishal ; FHI Solutions, Alive &Thrive<br>Escobar-Alegria , Jessica ; FHI Solutions, Alive &Thrive<br>Avula, Rasmi; International Food Policy Research Institute, Poverty, Health and Nutrition Division<br>Menon, Purnima; International Food Policy Research Institute, Poverty, Health and Nutrition Division |
| Keywords: | COVID-19, Nutrition < TROPICAL MEDICINE, Public health < INFECTIOUS DISEASES |
| | |

SCHOLARONE™
Manuscripts

For peer review only - http://bmjopen.bmj.com/site/about/guidelines.xhtml

*I, the Submitting Author has the right to grant and does grant on behalf of all authors of the Work (as defined in the below author licence), an exclusive licence and/or a non-exclusive licence for contributions from authors who are: i) UK Crown employees; ii) where BMJ has agreed a CC-BY licence shall apply, and/or iii) in accordance with the terms applicable for US Federal Government officers or employees acting as part of their official duties; on a worldwide, perpetual, irrevocable, royalty-free basis to BMJ Publishing Group Ltd ("BMJ") its licensees and where the relevant Journal is co-owned by BMJ to the co-owners of the Journal, to publish the Work in this journal and any other BMJ products and to exploit all rights, as set out in our licence.*

*The Submitting Author accepts and understands that any supply made under these terms is made by BMJ to the Submitting Author unless you are acting as an employee on behalf of your employer or a postgraduate student of an affiliated institution which is paying any applicable article publishing charge ("APC") for Open Access articles. Where the Submitting Author wishes to make the Work available on an Open Access basis (and intends to pay the relevant APC), the terms of reuse of such Open Access shall be governed by a Creative Commons licence – details of these licences and which Creative Commons licence will apply to this Work are set out in our licence referred to above.*

*Other than as permitted in any relevant BMJ Author's Self Archiving Policies, I confirm this Work has not been accepted for publication elsewhere, is not being considered for publication elsewhere and does not duplicate material already published. I confirm all authors consent to publication of this Work and authorise the granting of this licence.*

For peer review only

# The impact of COVID-19 on household food insecurity and interlinkages with child feeding practices and coping strategies in Uttar Pradesh, India

**Authors:**

Phuong Hong Nguyen[1], Shivani Kachwaha[1], Anjali Pant[1], Lan Mai Tran[2], Sebanti Ghosh[2], Praveen Kumar Sharma[2], Vishal Dev Shastri[2], Jessica Escobar-Alegria[2], Rasmi Avula[1], Purnima Menon[1]

**Author affiliations:**

[1] Poverty, Health and Nutrition Division, International Food Policy Research Institute, Washington, DC, USA; [2]Alive &Thrive, FHI Solutions, Washington, DC, USA

***Corresponding author:**

Phuong Hong Nguyen, Poverty, Health and Nutrition Division, International Food Policy Research Institute, 2005 I Street, NW, Washington DC, 20005. Phone: 202-826-4088. Email: P.H.Nguyen@cgiar.org

**Word count: 3458** (from introduction to conclusion, not including tables)

**Abbreviations**

AOR: : Adjusted odds ratio
COVID-19 : Coronavirus
HFI : Household food insecurity
PDS : Public distribution system
SES : Socio-economic status
THR : Take-home rations

For peer review only

## ABSTRACT

**Introduction**: The Coronavirus (COVID-19) pandemic has profound negative impacts on people's lives, but little is known on the effect of COVID-19 on household food insecurity (HFI) in poor setting resources. This study used digital data collection methods to assess the changes in HFI during the pandemic and examined the interlinkages between HFI with child feeding practices and coping strategies in Uttar Pradesh, India.

**Methods:** We conducted a longitudinal quantitative survey with 569 mothers with children <2y in December 2019 (in-person) and August 2020 (by phone). We measured HFI by using the Household Food Insecurity Access Scale and examined the changes in HFI during the pandemic using the Wilcoxon matched-pairs signed-rank tests. We then assessed child feeding practices and coping strategies by HFI status using multivariable regression models.

**Results**: HFI increased sharply from 21% to 80% before and during COVID-19, with 62% households changing the status from food security to insecurity and 17% remaining food insecure. Children belonging to newly and consistently food-insecure households were less likely to consume a diversified diet (adjusted odds ratio, AOR: 0.56, p=0.03 and AOR: 0.45, p=0.04, respectively) compared to those in food-secure households. Households with food insecurity were more likely to engage in coping strategies for obtaining foods including reducing other essential non-food expenditures (AOR: 1.7-2.2), borrowing money to buy food (AOR: 3.6- 4.3), selling jewelry (AOR: 3.0-5.0), and spending savings or selling other assets (AOR~2.0), all p<0.05.

**Conclusions:** COVID-19 had a significant negative impact on HFI, which in turn had implications for child feeding practices and coping strategies. Our findings demonstrated the feasibility of gathering information on HFI using digital data collection methods and highlighted the need for




For peer review only - http://bmjopen.bmj.com/site/about/guidelines.xhtml

further investment in targeted social protection strategies and safety nets as part of multisectoral solutions to improve HFI during and post-COVID-19.

**Keywords:** COVID-19, child feeding practices, coping strategies, household food insecurity, India, pandemic

For peer review only



For peer review only - http://bmjopen.bmj.com/site/about/guidelines.xhtml

**Strengths and limitations of this study**

- Using longitudinal surveys with a cohort of mothers with children <2 years, our study provides unique evidence of changes in household food insecurity before the pandemic and 6 months after the onset of COVID-19 in the context of a low-middle-income country.
- Our study bridges the gap in literature on the interlinkages between household food insecurity with child feeding practices and coping strategies to obtain foods to deal with household economic hardships during the pandemic.
- Our study demonstrates the feasibility of gathering information on household food insecurity via digital data collection methods but indicates some challenges including low response rate and inability to reach some of the poorest or most vulnerable households through phone surveys.
- Since all mothers in our study had children <6 months in December 2019, we were unable to obtain information on complementary feeding to compare child feeding practices before and during COVID pandemic.



For peer review only - http://bmjopen.bmj.com/site/about/guidelines.xhtml

## INTRODUCTION

The Coronavirus disease (COVID-19) has profound and wide-ranging public health impacts with significant global threat. Beyond the direct impacts from the virus, the pandemic will likely have a range of indirect consequences on food insecurity, child malnutrition, morbidity and mortality through disruptions in health and nutrition services, food supply chains, and livelihoods.[1-4] Early estimates suggest that potential disruptions of health systems and decreased access to food could lead to 1,157,000 additional child deaths and 56,700 additional maternal deaths.[5] Further, disruptions caused by the pandemic may affect households in multiple other ways including employment and income loss, mobility constraints, and household stress. Experts have warned about the potential consequences of COVID-19, ruining decades of progress, making it unlikely for low and middle income countries to reach the sustainable development goal to "end hunger, achieve food security and improved nutrition and promote sustainable agriculture" by 2030.[6]

There have been growing concerns on the impact of COVID-19 on household food insecurity (HFI).[7 8] Disruptions caused by the pandemic have the potential to influence all "four pillars" of food security including availability, access, utilization, and stability.[9] The pandemic may influence HFI directly on the supply side by disrupting food systems (such as primary food production, stability of food production, processing, food reserve stockpiles, and marketing) as well indirectly on the demand side due to impact of lockdowns on households' incomes, physical access to foods, and economic access to food.[10 11]

The impact of COVID-19 on HFI and poor health outcomes is complex, multilevel, and bidirectional.[4] At the household and individual levels, food insecurity is hypothesized to be a risk factor for both short- and long-term health outcomes through key three pathways: household stress (due to worrying about health issues, job loss and strained finances, and disconnection from social

For peer review only

support systems), behavioral coping mechanisms (engaging in high-risk behavior, compromise health care activities for foods, poor mental health and inadequate child feeding and nurturing), and inflammatory pathways.[4] Expected negative consequences on food, nutrition, and health security of vulnerable groups including young children, pregnant, and lactating women may further exacerbate existing social and health inequities.[12]

Despite established frameworks and global understanding of the threat to HFI during the pandemic, empirical investigations are very limited till date. India is facing the extreme levels of the double crises- COVID-19 and food insecurity,[13] carrying the second highest burden of COVID-19 in the world with nearly 8 million total confirmed cases and 119,502 deaths as of 28th October 2020.[14] Yet only few studies were available on food security using data at the farmer and supply-side level,[15, 16] and negligible evidence on the demand side. Very little is known about how women and children within households may be affected by HFI. Further, there is lack of empirical evidence on the changes in HFI from before and during the pandemic. Addressing this knowledge gap is critical for action, specifically at this decisive time in India when the COVID-19 trajectory is still uncertain, and there is growing concern about potential spikes in the coming months. Our study seeks to bridge this gap in the current literature with the objectives to 1) assess the changes in HFI before and during the pandemic in Uttar Pradesh, India; and 2) examine the interlinkages between HFI with child feeding practices and coping strategies to deal with household economic hardships and obtain foods. We also aim to demonstrate the feasibility of assessing HFI through digital data collection methods and potential implications from using this method.



For peer review only - http://bmjopen.bmj.com/site/about/guidelines.xhtml

# METHODS

## Design

This study is a follow up of a cluster-randomized trial (2017-2019) which aimed to assess the impact of strengthening delivery of maternal nutrition interventions, including micronutrient supplements and intensifying interpersonal counseling and community mobilization, implemented through government ANC services in Uttar Pradesh, India.[17] Details of the parent study have described elsewhere. Briefly, we conducted in-person repeated cross-sectional surveys of 1,800 recently delivered women as part of the cluster-randomized trial.[17] The end-line data collection was conducted in December 2019, prior to the onset of COVID-19 pandemic, providing an opportunity for a pre-and-post assessment of the effect of COVID-19 on food insecurity in this context.

## Data sources

The household survey was conducted with mothers of children <2 years old following the same study design and sampling frame as in the cluster-randomized trial. Of the 1,849 mothers survey at endline from the parent study in December 2019, 587 could be reached for a phone interview in August 2020, yielding a response rate of 32% **(Figure 1).** Reasons for not being able to conduct phone survey included unavailable contact phone number (n=388), phone unreachable or switched off (n=667), wrong number (n=136), refusal to participate (n=63) and child death (n=9). Reasons for losses to follow up in phone survey were similar between intervention and comparison areas (results not shown). The total sample of non-pregnant mothers (n=569) who were interviewed in both surveys were used for the analysis.

**<Insert Figure 1 here>**



For peer review only - http://bmjopen.bmj.com/site/about/guidelines.xhtml

**Variables**

Household food security was measured before and during the pandemic using the standard FANTA/USAID's Household Food Insecurity Access Scale.[18] Mothers were asked nine questions related to the household's experience of food insecurity in the 30 days preceding the survey. These questions capture 3 main domains of household food insecurity: anxiety and uncertainty about the household food supply (1 item), insufficient quality (3 items), and insufficient quantity and its physical consequences (5 items). We reported the percentage of households that experienced 1) any food insecurity occurrence among nine questions, 2) any of a specific domain, and 3) food insecurity condition categorized as food-secure and mild, moderately, or severely food-insecure.

Information on child feeding practices were collected using the open 24hr dietary recall, where the mother was asked about all foods and liquids consumed by the child in different time periods of the previous 24 hours before the survey. All food items were categorized into the 7 food groups used in the WHO guideline:[19] 1) starchy staple foods, 2) legumes and nuts, 3) dairy products (milk, yogurt, and cheese), 4) flesh foods, 5) eggs, 6) vitamin A rich fruits and vegetables, and 7) other fruits and vegetables. Minimum dietary diversity was defined as children who consumed foods from 4 or more out of 7 food groups in the previous 24 hours. Data for complementary feeding practices were not available during the in-person survey in December 2019, because all mothers had children <6 months during that time.

Households were also asked about access to social protection, especially food supplementation they received for mothers and children from the government during the lockdown period and during the 30 days prior to the survey, such as take-home rations (THR) and use of public distribution system (PDS). Finally, information on different coping strategies that the household had



For peer review only - http://bmjopen.bmj.com/site/about/guidelines.xhtml

to engage in the past 30 days due to lack of food was collected, including spending savings, reducing essential non-food expenditure, borrowing money, or selling jewelry/gold.

Other potential factors associated with food security or child feeding practices were obtained at maternal (age, education level, and occupation), child (age and sex), and household levels (religion, scheduled caste/tribal - designated historically disadvantaged groups in India, number of children <5y, and household socio-economic status- SES). The SES index was constructed using a principal component analysis from multiple variables including household ownership of assets, livestock, and housing quality.[20]

**Data analysis**

We compared background characteristics of the analytic sample (mothers who completed both surveys, in-person survey before COVID and phone survey during COVID) and the non-analytic sample (those who completed in-person surveys only) using student t-test (for continuous variables) and chi-square test (for categorical variables). We used descriptive analysis to report HFI and child feeding practices. We examined changes in HFI before and during the pandemic using Wilcoxon matched-pairs signed-rank tests.

To examine differences in child feeding practices and coping strategies by food insecurity status, we created three categories of households: 1) food secure (households who were food secure before and during COVID-19 pandemic and those who were food insecure at some point before but were no longer food insecure during the pandemic), 2) consistently food insecure (both food insecure before and during COVID-19); 3) newly food insecure (food secure before COVID-19 but became food insecure during the pandemic). We then compared child feeding practices and coping strategies among the three categories using multivariable regression models, adjusting for child age and sex, mother's age, religion, and education, scheduled caste, and number of children <5y in the



For peer review only - http://bmjopen.bmj.com/site/about/guidelines.xhtml

household. We also examined uptake of social protection programs such as food supplementation and cash transfer as potential strategies to improve HFIs. All statistical analyses were undertaken using Stata version 16.

**Ethical approval**

Informed consent in local language was obtained from mothers, FLWs, and block managers prior to their participation in the study. The research protocol received ethical clearance from the Institutional Review Board at the International Food Policy Research Institute (IRB #00007490) and the Suraksha Independent Ethics Committee in India (IRB #2017-10-9094). Additional permissions for data collection were provided by State Government of Uttar Pradesh.

**Patient and public involvement statement**

Patients or the public were not involved in the design, or conduct, or reporting, or dissemination plans of our research

# RESULTS

**Characteristics of the study sample**

At the time of in-person survey in December 2019, all mothers had an infant between the ages of 0–5.9 months of age with an average age of 3 months (Table 1). On average, mothers were ~26 years and majority of them (>90%) were housewives. Nearly all women were Hindu (92%) and nearly half of them belonged to a backward community (44-47%). Mothers in the final analytic sample had higher education (8.2 vs. 6.7 years of schooling) and lived in wealthier (27% vs. 17% in quintile 5) and more food secure households (79% vs. 75%) compared to those in non-analytic sample. Mothers belonging to intervention and control areas of the maternal nutrition intervention (from 2017 to 2019) were equally represented in the analytic sample.



For peer review only - http://bmjopen.bmj.com/site/about/guidelines.xhtml

**Table 1: Background characteristics[1] of the study sample participated in surveys before and during the COVID pandemic (December 2019 and August 2020)**

| | Analytic sample (both in person and phone surveys before and during the pandemic) (n= 569) | Non-analytic sample (only in person survey before the pandemic) (n= 1,280) | p |
|---|---|---|---|
| Age of respondent mother (years) | 25.5 (3.8) | 25.7 (4.0) | 0.47 |
| Education (years) | 8.2 (4.3) | 6.7 (4.6) | <0.001 |
| Never attended school | 14.1 | 24.8 | <0.001 |
| Primary school (grade1-5) | 13.9 | 16.3 | |
| Middle school (grade 6-9) | 24.3 | 24.7 | |
| High school (grade 10-12) | 30.1 | 23.3 | |
| Graduate and above | 17.8 | 10.9 | |
| Occupation as housewife | 91.7 | 93.0 | 0.35 |
| Child age, mos | 3.0 (1.6) | 2.8 (1.6) | 0.041 |
| Child sex (male) | 49.0 | 49.5 | 0.84 |
| Number of children <5y | 1.6 (0.7) | 1.7 (0.7) | 0.60 |
| Religion as Hindu | 93.7 | 91.1 | 0.061 |
| Caste category | | | |
| Scheduled caste/tribe | 38.3 | 38.4 | 0.25 |
| Other Backward Class | 44.3 | 47.0 | |
| General/others | 17.4 | 14.5 | |
| Household socio-economic status | | | |
| Quintile 1 | 11.6 | 23.8 | <0.001 |
| Quintile 2 | 19.2 | 20.4 | |
| Quintile 3 | 18.1 | 20.9 | |
| Quintile 4 | 24.6 | 18.0 | |
| Quintile 5 | 26.5 | 17.0 | |
| Household food security status | | | |
| Food secure | 79.3 | 74.5 | 0.08 |
| Mildly food insecure | 5.6 | 5.9 | |
| Moderate food insecure | 5.1 | 5.3 | |
| Severe food insecure | 10.0 | 14.3 | |
| Maternal nutrition (2017-2019) | | | |
| Intervention area | 282 | 640 | |
| Comparison area | 287 | 640 | |

[1]Background data presented in this table were from in -person survey in December 2019

For peer review only



For peer review only - http://bmjopen.bmj.com/site/about/guidelines.xhtml

## Changes in food security status during the COVID pandemic

Prior to the pandemic, 21% of households were identified as food insecure. During the pandemic, HFI experiences sharply increased for each separate item and each domain, as well as among the categories (Figure 2). For example, the prevalence of anxiety and uncertainty about the household food supply, insufficient quality of food, and insufficient quantity of food consumed during the pandemic were 45%, 78%, and 42%, respectively, which was much higher than before the pandemic (12%, 18%, and 14%, respectively). The prevalence of any food insecurity increased from 21% to 80%, of which mildly, moderately and severely food insecure households increased by 14 percentage points (pp), 25 pp and 20 pp, respectively. Overall, 62% households changed from being food secure to insecure during the pandemic, while only 17% remained food insecure.

**<Insert Figure 2 here>**

## Child feeding practices during the COVID pandemic

Child feeding practices is of a major concern, with only 19% of children achieved minimum dietary diversity (≥ 4 food groups). Extremely low proportion of children were fed with flesh food (1%), egg (1%) and vitamin-A rich fruit and vegetables (4%). A third of children consumed other fruits and vegetables and nearly two thirds consumes legumes and nuts in the 24 hours prior to the survey (Figure 3).

**<Insert Figure 3 here>**

## Association between food insecurity status and child feeding practices during the COVID pandemic

Children living in households that became food-insecure since the pandemic were less likely to consume a diversified diet (18% vs. 28%; adjusted odds ratio, AOR: 0.56, p= 0.025) as well as legumes and nuts (57% vs. 69%; AOR: 0.59, p= 0.024) compared to children living in consistently



For peer review only - http://bmjopen.bmj.com/site/about/guidelines.xhtml

food secure households (Table 2). The child feeding practices were worse in the households that were food insecure at both times. Specifically, fewer children in consistently food-insecure households consumed a diverse diet (12.4% vs. 28%; AOR: 0.45, p= 0.044) and other fruits and vegetables (21% vs. 40%; AOR: 0.47, p= 0.021) compared to those in food secure households.

**Table 2: Association between child dietary diversity and household food insecurity status during the pandemic**

| | Currently food secure | Newly food insecure[1] | Consistent food insecurity | New food insecurity vs. food secure[2] | | Consistent food insecurity vs. food secure[2] | |
|---|---|---|---|---|---|---|---|
| | n=116 | n=354 | n=99 | | | | |
| | % | % | % | OR (95%CI) | p | OR (95%CI) | p |
| Grain | 79.3 | 80.8 | 78.8 | 0.98 (0.57, 1.67) | 0.93 | 0.85 (0.42, 1.70) | 0.64 |
| Legumes and nuts | 69.0 | 56.8 | 55.6 | 0.59 (0.37, 0.93) | 0.024 | 0.62 (0.35, 1.12) | 0.11 |
| Dairy | 74.1 | 76.3 | 79.8 | 1.20 (0.73, 1.96) | 0.47 | 1.63 (0.83, 3.19) | 0.16 |
| Flesh foods | 0.9 | 0.6 | 2.0 | 0.31 (0.04, 2.30) | 0.25 | 0.72 (0.08, 6.55) | 0.77 |
| Eggs | 0.9 | 1.1 | 1.0 | 1.31 (0.14, 12.25) | 0.82 | 1.18 (0.07, 21.38) | 0.91 |
| Vit A rich fruits and vegetables | 4.3 | 4.3 | 2.0 | 0.87 (0.30, 2.50) | 0.79 | 0.43 (0.08, 2.36) | 0.33 |
| Other fruits and vegetables | 39.7 | 33.5 | 21.2 | 0.73 (0.46, 1.14) | 0.17 | 0.47 (0.24, 0.89) | 0.021 |
| Minimum dietary diversity (≥ 4 food groups) | 28.1 | 17.9 | 12.4 | 0.56 (0.33, 0.93) | 0.025 | 0.45 (0.21, 0.98) | 0.044 |

[1]Currently food secure was defined as households who were food secure before and during COVID-19 pandemic and those who were food insecure at some point before but were no longer food insecure during the pandemic, consistent food insecurity was defined as both food insecure before and during COVID-19; newly food insecurity insecure was defined as food secure before COVID-19 but became food insecure during the pandemic. [2]Model controlled for child age, sex, mother's age, education caste, religion, and number of children<5y in the household.

For peer review only

**Challenges faced during the pandemic**

The key challenges faced by households in consuming food in the last 7 days preceding the survey include non-availability of funds to buy food (59%), non-availability of foods in market area (21%), increase in food prices (17%), and inability to travel or transport issues (21%). The pandemic resulted in unemployment/loss of income in 78.4% households.

**Coping strategies and household food insecurity status during the COVID pandemic**

More than 60% households disbursed their savings and reduced their expenses on health and non-food essentials to meet food and other requirements, irrespective of their food security status (Table 3). Households experiencing food insecurity were more likely to engage in coping strategies related to obtaining food including reducing their expenditure on non-food essentials (AOR: 1.7 and 2.2 for newly an consistently food-insecure households, respectively), borrowing money to buy food (AOR: 3.6 and 4.3, respectively), and selling jewelry (AOR: 3.0 and 5.0, respectively). Additionally, newly food-insecure households were ~2 times more likely to spend saving or sell households/assets/transport means.



For peer review only - http://bmjopen.bmj.com/site/about/guidelines.xhtml

**Table 3: Association between current coping strategies and household food insecurity status during the pandemic**

| Indicators | Currently food secure | Newly food insecure[1] | Consistent food insecurity | New food insecurity vs. food secure[2] | | Consistent food insecurity vs. food secure[2] | |
|---|---|---|---|---|---|---|---|
| | n=116 | n=354 | n=99 | | | | |
| | % | % | % | OR [95%CI] | p | OR [95%CI] | p |
| Spent savings | 83.6 | 91.0 | 89.9 | 2.05 (1.09, 3.88) | 0.027 | 1.73 (0.71, 4.18) | 0.23 |
| Reduced health expenditure | 64.7 | 72.0 | 74.7 | 1.33 (0.84, 2.10) | 0.23 | 1.49 (0.79, 2.80) | 0.22 |
| Reduced other essential non-food expenditures such as education and clothes | 66.4 | 77.4 | 81.8 | 1.73 (1.08, 2.78) | 0.024 | 2.15 (1.09, 4.24) | 0.027 |
| Borrowed money to buy food | 25.0 | 54.8 | 63.6 | 3.57 (2.19, 5.80) | <0.001 | 4.29 (2.31, 7.95) | <0.001 |
| Reduced expenses on agricultural, livestock or fisheries inputs | 23.3 | 33.3 | 35.4 | 1.64 (0.99, 2.72) | 0.055 | 1.78 (0.94, 3.38) | 0.078 |
| Selling jewellery/gold | 4.3 | 13.0 | 21.2 | 3.03 (1.16, 7.92) | 0.024 | 4.98 (1.74, 14.27) | 0.003 |
| Selling household goods or productive assets or means of transport | 19.0 | 29.4 | 27.3 | 1.78 (1.03, 3.07) | 0.038 | 1.64 (0.83, 3.26) | 0.16 |

[1]Currently food secure was defined as households who were food secure before and during COVID-19 pandemic and those who were food insecure at some point before but were no longer food insecure during the pandemic, consistent food insecurity was defined as both food insecure before and during COVID-19; newly food insecurity insecure was defined as food secure before COVID-19 but became food insecure during the pandemic. [2]Model controlled for child age, sex, mother's age, education caste, religion, and number of children<5y in the household.

**Social protection before and during the COVID pandemic**

The proportion of households receiving THR was similar before and during the pandemic, with slightly higher in food-insecure (~63%) compared to food-secure households (55-59%) (Figure 4). In contrast, coverage of PDS ration increased significantly during the pandemic for both food-insecure (61% to 71%) and food-secure households (from 49% to 72%); the increase was smaller among beneficiaries from consistently food-insecure compared to those in food-secure households (9.3 pp vs. 23 pp).

**<Insert Figure 4 here>**

For peer review only



For peer review only - http://bmjopen.bmj.com/site/about/guidelines.xhtml

## DISCUSSION

In response to global concerns on the impact of COVID-19 on maternal and child food and nutrition insecurity, our study provides unique evidence of changes in HFI before and during the pandemic and its linkage with child feeding practices as well as coping strategies to obtain foods among food secure and insecure households. We found that HFI increased substantially during the pandemic (60 pp), with a large portion related to insufficient quality (78%) and lower levels related to insufficient quantity (42%). Children living in food-insecure households were less likely to consume a diversified diet, mainly due to less consumption of legumes and nuts, fruits and vegetables. In order to overcome the challenges during the pandemic, households were compelled to engage in several coping strategies related to spending existing savings, reducing household expenditures, selling assets, or borrowing money.

Our findings were consistent with global literature on the increase in HFI during the pandemic.[21-25] However, most previous studies were conducted in developed countries and mainly obtained information during the pandemic. The only two studies with information before and during COVID-19 time was from US, one found 32% increase in HFI since COVID-19 [26] while the other found an increase of 20%.[21] Our study showed much higher magnitude of increase in HFI (~60%), which is a worrisome finding given the high pre-existing levels of food insecurity in India. We also found that HFI was predominantly due to insufficient food quality concerns which was aligned with a previous study which showed increased consumption of high-calorie snack foods and sweets,[21 23] or cheaper highly processed foods.[4]

Our findings indicate challenges to several food security dimensions, including livelihood and income loss, economic and physical access, availability, and utilization. A study on livelihood and dietary effects of COVID-19 with vegetable producers in four states of India reported negative impacts on production, sales, prices, and incomes among majority of farmers,[15] Farm households



For peer review only - http://bmjopen.bmj.com/site/about/guidelines.xhtml

also reported disruptions to their diets with reduced ability to access nutrient-dense foods, particularly fruit and animal source foods.[15] Another study in Maharashtra, India finds disruptions in the urban-rural food supply chain due to the closure of wholesale markets with uncertainties in food supply, declines in market availability, and increase in food prices.[16] These findings are complementary to our study and the supply-side insights possibly explain some of the trends we observe in food security, child feeding, and coping strategies.

To our knowledge, infant and child feeding practices during the pandemic have not been explored in the literature. Our findings showed that the diets of children were suboptimal, with only 19% achieving minimum dietary diversity – a similar result compared to a previous study in Uttar Pradesh, India before COVID pandemic (17%).[27] We also found that children living in food-insecure households had much poorer diets than those in food-secure household, but the proportion of children consuming flesh foods, eggs, and vitamin A fruits and vegetables is very low, irrespective of food security status. During the COVID-19 pandemic, child feeding practices has been reported to change, particularly among food-insecure households, due to higher levels of stress, fewer resources, and less access to food and affordability, leading to restrict the quantity and quality of food their children eat and more parents' controlling feeding behaviors.[21] Other studies also showed that mothers of the children in food-insecure households often prioritized shelf-stable foods to deal with food supply disruptions and social-distancing policies, and have tendency to rely on energy-dense foods for a longer period of time.[4]

We found that all households in our study engaged in some coping strategies to obtain food regardless of HFI status, but food-insecure households were more likely to engage in several such practices. Our findings are consistent with literature stating that the main strategies that food-insecure households generally rely on to maintain access to food include shifting within their own



For peer review only
For peer review only - http://bmjopen.bmj.com/site/about/guidelines.xhtml

spending patterns to prioritize food (reducing expenses on health, other non-food expenditures, or agricultural, livestock or fisheries inputs), relying on social network, or access to government nutrition programs.[4 26] However, all these strategies can easily be impacted when COVID pandemic severely affects the entire household budget, or social-distancing policies could affect network access. The coping strategies households have adopted to obtain food during COVID-19 will run out and will not suffice for preventing HFI from getting worse if the pandemic crisis continues.

Social protection strategies are an important intervention to address the rising levels of HFI in the context of COVID-19, particularly for low-income countries.[28] A global review of evidence by World Bank found that India increased coverage of cash transfers from ~2% before the pandemic to about 15% during COVID.[29] The Indian government also initiated home-delivery of take-home rations for pregnant and lactating women and children and provided one-month free supply of wheat and rice to the poorest ration card holders through the public distribution system.[30] Our findings on the increased access to PDS during COVID-19 align with previous conclusions about the important role of the program as an essential component of the Government's response to food insecurity.[31] Despite these measures, food supplementation was received among just over half of households and the increase in access to PDS was smaller among beneficiaries that are consistently food insecure compared to those food secure. These results highlights an important opportunity to strengthen the Government's response to reduce food insecurity during and after COVID-19 in the short term by improving efficiency of existing social protection strategies and targeting to the most vulnerable populations.[31 32] Other strategies which may be considered include outlining specific recommendations to ensure food security for poor and vulnerable populations as done for other developing countries in Africa [33] and include special initiatives for migrant populations.[34] Certain agricultural reforms may also be considered[35] such as home gardening,[36] diversification of



For peer review only - http://bmjopen.bmj.com/site/about/guidelines.xhtml

production, and strong local market chains [37] to alleviate HFI, improve diets, and reduce reliance on coping strategies due to food insecurity.

Our study followed the cohort of mothers before the pandemic and 6 months after the onset of COVID-19, thus offered a unique and timely contribution to the literature on the magnitude and nature of increase in HFI before and during the pandemic, and its implications for child feeding practices and coping strategies in the context of a low-middle-income country with prevailing high HFI. Given the restrictions on movement and contacting people, we were able to mobilize phone survey to reach mothers and using the same instrument to measure food security over time. Our experience demonstrated the feasibility of gathering information on HFI via digital data collection methods but indicated potential challenges and bias in the background characteristics of respondents interviewed through in-person vs. phone surveys. Mothers who responded to phone survey had slightly higher education and SES background compared to those in the non-analytic sample (only in-person survey), indicated that we may not be able to reach some of the poorest or most vulnerable households through the phone survey. We also experienced similar challenges as other phone surveys[38] including low response rate, several calling schedules during the survey and potential unknown response bias. Finally, since all mothers in our study had children <6 months in December 2019, we were unable to obtain information on complementary feeding to compare child feeding practices before and during COVID time.

## CONCLUSION

COVID-19 had a significant negative impact on HFI in this context, which in turn had implications for child feeding practices and reliance on coping strategies to obtain foods. Our study highlighted the opportunity to reduce HFI in the short-term with existing resources by improving the



For peer review only - http://bmjopen.bmj.com/site/about/guidelines.xhtml

targeting of social protection benefits to effectively reach the food insecure and make quality diets accessible. Given the great concern of the expected increase in HFI as the pandemic continues, strengthened multisectoral response is needed to ensure effective re-establishment of health and nutrition services, food supply chains, and restoration of livelihoods to improve household food security during and after the pandemic.

For peer review only



For peer review only - http://bmjopen.bmj.com/site/about/guidelines.xhtml

## FIGURE LEGENDS

**Figure 1.** Participant flow

**Figure 2.** Food insecurity experienced by mothers and their household members in the previous 30 days before and during the COVID pandemic

**Figure 3:** Child feeding practices during the COVID pandemic

**Figure 4:** Household receipt of social protection benefits before and during the pandemic, by household food insecurity status

For peer review only

## AUTHOR CONTRIBUTIONS

PHN: conceive paper, analysis, draft manuscript, consolidate comments from all co-authors, revised and finalize paper.

SK: Field work coordination, literature review, draft some parts of the manuscript, revised and finalize paper.

AP: Field work coordination, data analyses, draft some parts of the manuscript, review manuscript.

LMT: data analyses, visualization for data presentation, review manuscript.

SG, PKS, VDS, JE: data interpretation and its implications, reviewed and edited the manuscript.

RA and PM reviewed the statistical analyses, supported data interpretation, reviewed and edited the manuscript.

All authors read and approved the final submitted manuscript.

## SOURCE OF FUNDING:

Bill & Melinda Gates Foundation through POSHAN, led by International Food Policy Research Institute. Grant number: OPP50838.

## CONFLICT OF INTEREST STATEMENT:

Authors declare no conflicts of interest.

## DATA AVAILABILITY STATEMENT:

All relevant data are within the manuscript and its Supporting Information files. Additional original data can be provided upon request.



For peer review only - http://bmjopen.bmj.com/site/about/guidelines.xhtml

## REFERENCES

1. Kempf AM, Remington PL. New challenges for telephone survey research in the twenty-first century. *Annu Rev Public Health* 2007;28:113-26. doi: 10.1146/annurev.publhealth.28.021406.144059 [published Online First: 2006/11/11]
2. Tesfaye A, Habte Y, Minten B. COVID-19 is shifting consumption and disrupting dairy value chains in Ethiopia. IFPRI book chapters, in: COVID-19 and global food security, chapter 9, pages 42-45, International Food Policy Research Institute (IFPRI). 2020
3. Hirvonen K, De Brauw A, Abate G. Food Consumption and Food Security during the COVID-19 Pandemic in Addis Ababa. IFPRI Discussion Paper 01964. 2020
4. Headey D, Heidkamp R, Osendarp S, et al. Impacts of COVID-19 on childhood malnutrition and nutrition-related mortality. *Lancet* 2020;396(10250):519-21. doi: 10.1016/S0140-6736(20)31647-0 [published Online First: 2020/07/31]
5. Leddy AM, Weiser SD, Palar K, et al. A conceptual model for understanding the rapid COVID-19-related increase in food insecurity and its impact on health and healthcare. *Am J Clin Nutr* 2020 doi: 10.1093/ajcn/nqaa226 [published Online First: 2020/08/09]
6. Roberton T, Carter ED, Chou VB, et al. Early estimates of the indirect effects of the COVID-19 pandemic on maternal and child mortality in low-income and middle-income countries: a modelling study. *Lancet Glob Health* 2020;8(7):e901-e08. doi: 10.1016/S2214-109X(20)30229-1 [published Online First: 2020/05/15]
7. United Nations. Resolution adopted by the General Assembly on 25 September 2015, Transforming our world: the 2030 Agenda for Sustainable Development (A/RES/70/1) 2015
8. Paslakis G, Dimitropoulos G, Katzman DK. A call to action to address COVID-19-induced global food insecurity to prevent hunger, malnutrition, and eating pathology. *Nutr Rev* 2020 doi: 10.1093/nutrit/nuaa069 [published Online First: 2020/07/12]
9. Cable J, Jaykus LA, Hoelzer K, et al. The impact of COVID-19 on food systems, safety, and security-a symposium report. *Ann N Y Acad Sci* 2020 doi: 10.1111/nyas.14482 [published Online First: 2020/08/30]
10. Laborde D, Martin W, Swinnen J, et al. COVID-19 risks to global food security. *Science* 2020;369(6503):500-02. doi: 10.1126/science.abc4765 [published Online First: 2020/08/01]
11. Devereux S, Bene C, Hoddinott J. Conceptualising COVID-19's impacts on household food security. *Food Secur* 2020:1-4. doi: 10.1007/s12571-020-01085-0 [published Online First: 2020/08/25]
12. Savary S, Akter S, Almekinders C, et al. Mapping disruption and resilience mechanisms in food systems. *Food Secur* 2020:1-23. doi: 10.1007/s12571-020-01093-0 [published Online First: 2020/08/25]
13. Perez-Escamilla R, Cunningham K, Moran VH. COVID-19 and maternal and child food and nutrition insecurity: a complex syndemic. *Matern Child Nutr* 2020;16(3):e13036. doi: 10.1111/mcn.13036 [published Online First: 2020/05/28]
14. Mishra K, Rampal J. The COVID-19 pandemic and food insecurity: A viewpoint on India. *World Development* https://doiorg/101016/jworlddev2020105068 2020;135:1-3.
15. Ministry of Health and Family Welfare. COVID-19 INDIA as on : 14 October 2020, 08:00 IST (GMT+5:30) New Delhi, India: Government of India; 2020 [Available from:



For peer review only - http://bmjopen.bmj.com/site/about/guidelines.xhtml

https://www.mohfw.gov.in/index1.php?lang=1level=1sublinkid=6471lid=4270 accessed 15th October 2020.
16. Harris J, Depenbusch L, Pal AA, et al. Food system disruption: initial livelihood and dietary effects of COVID-19 on vegetable producers in India. *Food Secur* 2020:1-11. doi: 10.1007/s12571-020-01064-5
17. Sukhwani V, Deshkar S, Shaw R. COVID-19 Lockdown, Food Systems and Urban-Rural Partnership: Case of Nagpur, India. *Int J Environ Res Public Health* 2020;17(16) doi: 10.3390/ijerph17165710 [published Online First: 2020/08/14]
18. Nguyen P, Kachwaha S, Tran LM, et al. Strengthening Nutrition Interventions in Antenatal Care Services Had Modest Impacts on Diet Diversity, Micronutrient Intake, and Breastfeeding in Uttar Pradesh, India. *Current Developments in Nutrition,* 2020;4(Supplement_2):1050, https://doi.org/10.93/cdn/nzaa054_122.
19. Coates J, Swindale A, Bilinsky P. Household Food Insecurity Access Scale (HFIAS) for Measurement of Household Food Access: Indicator Guide (v. 3). Washington, D.C.: Food and Nutrition Technical Assistance Project, Academy for Educational Development. 2007
20. WHO. Indicators for assessing infant and young child feeding practices. Part 2: Measurements. http://whqlibdoc.who.int/publications/2010/9789241599290_eng.pdf. World Health Organization: Geneva, 2008.
21. Filmer D, Pritchett LH. Estimating wealth effects without expenditure data--or tears: an application to educational enrollments in states of India. *Demography* 2001;38(1):115-32. [published Online First: 2001/03/03]
22. Adams EL, Caccavale LJ, Smith D, et al. Food insecurity, the home food environment, and parent feeding practices in the era of COVID-19. *Obesity (Silver Spring)* 2020 doi: 10.1002/oby.22996 [published Online First: 2020/08/08]
23. Bene C. Resilience of local food systems and links to food security - A review of some important concepts in the context of COVID-19 and other shocks. *Food Secur* 2020:1-18. doi: 10.1007/s12571-020-01076-1 [published Online First: 2020/08/25]
24. Carroll N, Sadowski A, Laila A, et al. The Impact of COVID-19 on Health Behavior, Stress, Financial and Food Security among Middle to High Income Canadian Families with Young Children. *Nutrients* 2020;12(8) doi: 10.3390/nu12082352 [published Online First: 2020/08/14]
25. Kent K, Murray S, Penrose B, et al. Prevalence and Socio-Demographic Predictors of Food Insecurity in Australia during the COVID-19 Pandemic. *Nutrients* 2020;12(9) doi: 10.3390/nu12092682 [published Online First: 2020/09/06]
26. Owens MR, Brito-Silva F, Kirkland T, et al. Prevalence and Social Determinants of Food Insecurity among College Students during the COVID-19 Pandemic. *Nutrients* 2020;12(9) doi: 10.3390/nu12092515 [published Online First: 2020/08/23]
27. Niles MT, Bertmann F, Belarmino EH, et al. The Early Food Insecurity Impacts of COVID-19. *Nutrients* 2020;12(7) doi: 10.3390/nu12072096 [published Online First: 2020/07/19]
28. MoHFW. Ministry of Health and Family Welfare (MoHFW), Government of India, UNICEF and Population Council. Comprehensive National Nutrition Survey (CNNS) National Report. New Delhi, India, 2019.
29. Pereira M, Oliveira AM. Poverty and food insecurity can increase as threats of COVID-19 spreads. *Public Health Nutr* 2020:1-10. doi: 10.1017/S1368980020003493 [published Online First: 2020/09/09]



For peer review only - http://bmjopen.bmj.com/site/about/guidelines.xhtml

30. Gentilini U, Almenfi M, Orton I, et al. Social Protection and Jobs Responses to COVID-19 : A Real-Time Review of Country Measures. Washington DC: World Bank, 2020.
31. International Food Policy Research Institute. POSHAN COVID-19 Resources: Online archive for COVID-19 related resources on nutrition, early childhood development and food security New Delhi, India: POSHAN; 2020 [Available from: https://poshancovid19.in/Resources.html accessed 15th October 2020.
32. George NA, McKay FH. The Public Distribution System and Food Security in India. *Int J Environ Res Public Health* 2019;16(17) doi: 10.3390/ijerph16173221 [published Online First: 2019/09/06]
33. FAO, IFAD, UNICEF, et al. The State of Food Security and Nutrition in the World 2020. Transforming food systems for affordable healthy diets. Rome, FAO. DOI: https://doi.org/10.4060/ca9692en. 2020
34. Lawson-Lartego L, Cohen MJ. 10 recommendations for African governments to ensure food security for poor and vulnerable populations during COVID-19. *Food Secur* 2020;12(4):899-902. doi: 10.1007/s12571-020-01062-7 [published Online First: 2020/08/26]
35. Smith MD, Wesselbaum D. COVID-19, Food Insecurity, and Migration. *J Nutr* 2020 doi: 10.1093/jn/nxaa270 [published Online First: 2020/08/26]
36. Kumar A, Padhee AK, Kumar S. How Indian agriculture should change after COVID-19. *Food Secur* 2020:1-4. doi: 10.1007/s12571-020-01063-6 [published Online First: 2020/08/25]
37. Lal R. Home gardening and urban agriculture for advancing food and nutritional security in response to the COVID-19 pandemic. *Food Secur* 2020:1-6. doi: 10.1007/s12571-020-01058-3 [published Online First: 2020/08/25]
38. Heck S, Campos H, Barker I, et al. Resilient agri-food systems for nutrition amidst COVID-19: evidence and lessons from food-based approaches to overcome micronutrient deficiency and rebuild livelihoods after crises. *Food Security* *https://doiorg/101007/s12571-020-01067-2* 2020

For peer review only



For peer review only - http://bmjopen.bmj.com/site/about/guidelines.xhtml

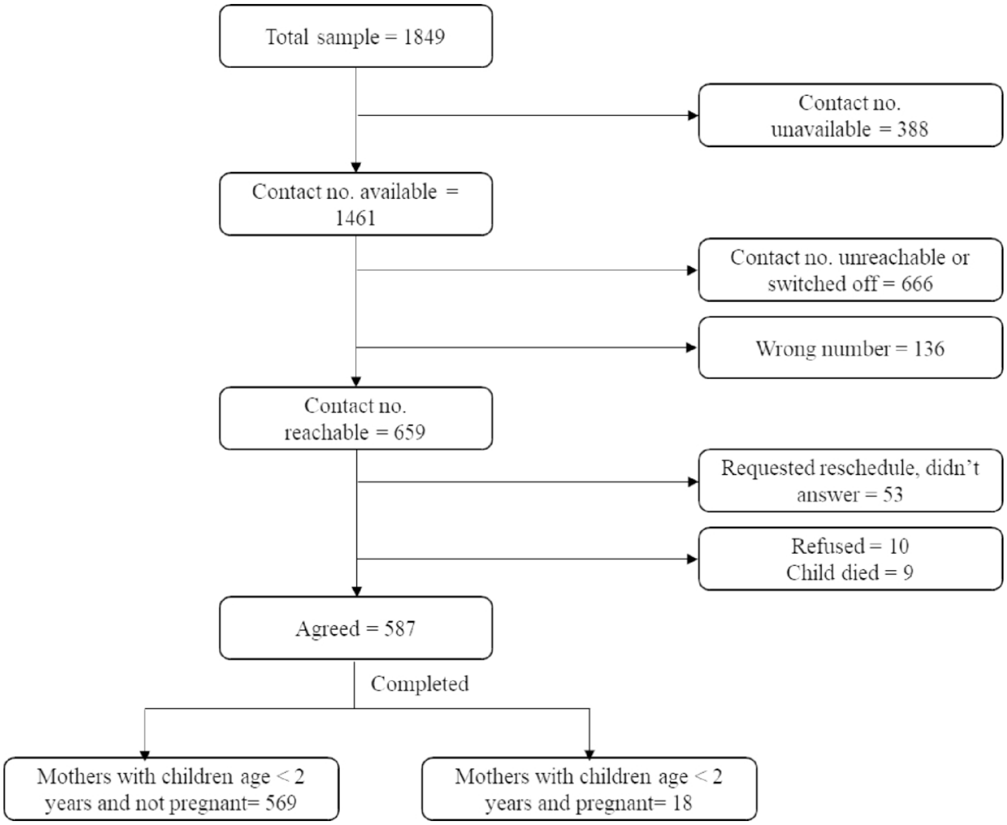


Figure 1. Participant flow

172x140mm (300 x 300 DPI)

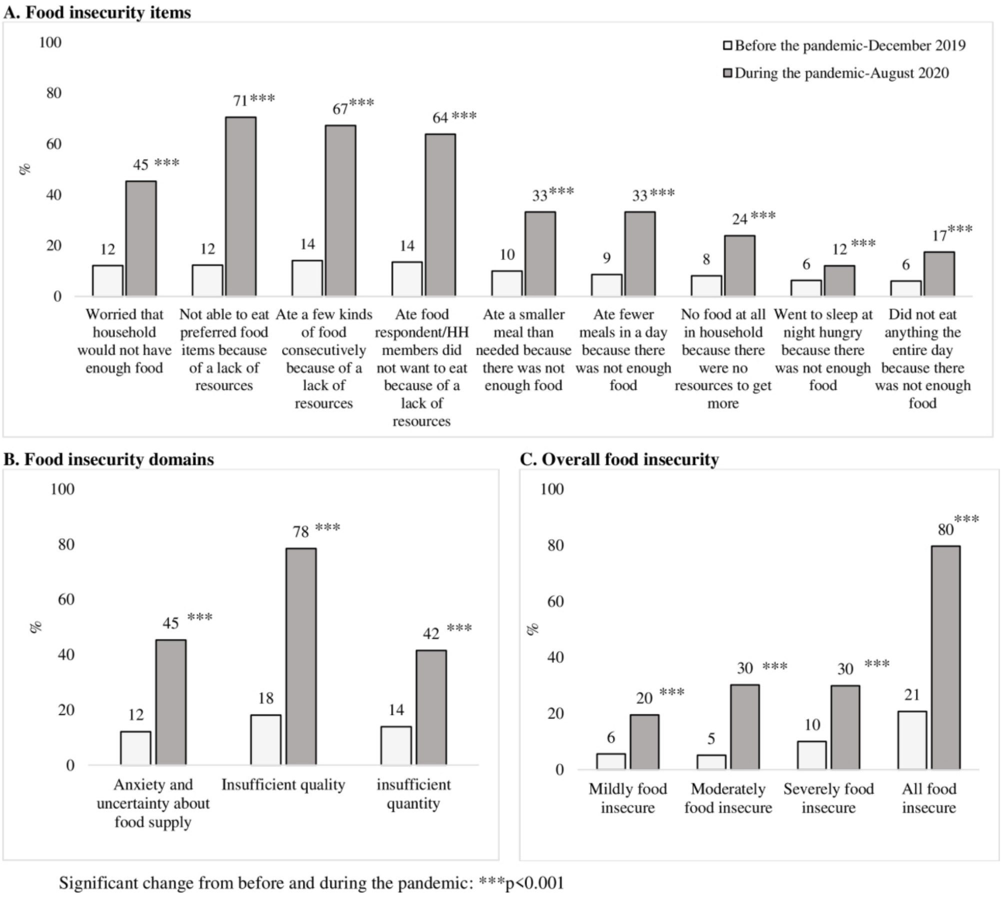


Figure 2. Food insecurity experienced by mothers and their household members in the previous 30 days before and during the COVID pandemic

190x168mm (300 x 300 DPI)

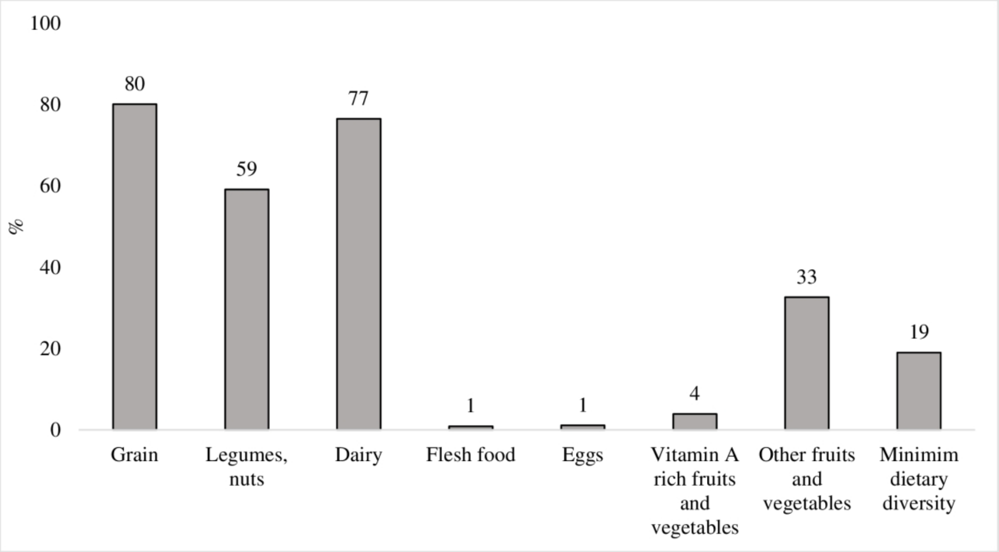


Figure 3: Child feeding practices during the COVID pandemic

168x92mm (300 x 300 DPI)

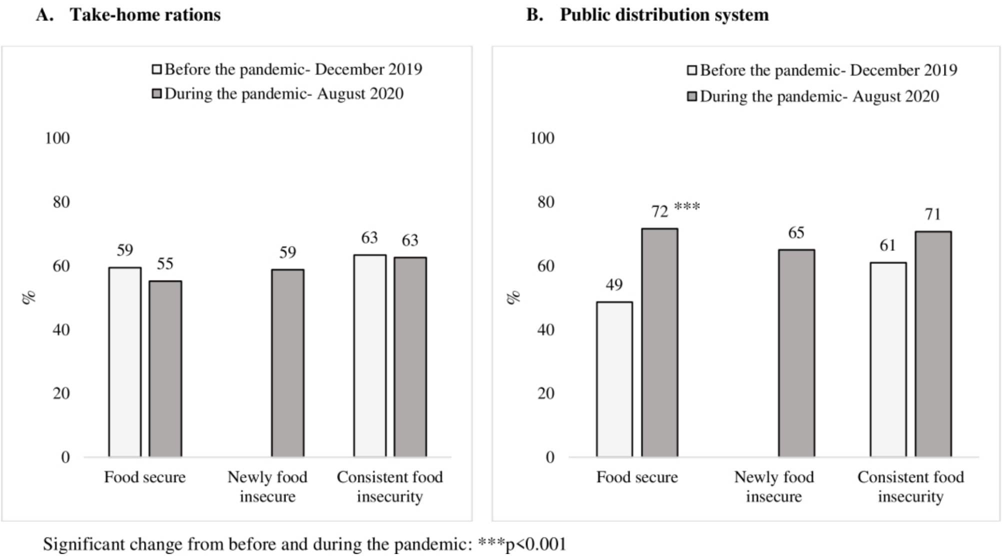


Figure 4: Household receipt of social protection benefits before and during the pandemic, by household food insecurity status

187x102mm (300 x 300 DPI)

## STROBE Statement—Checklist of items that should be included in reports of cross-sectional studies

| | Item No | Recommendation | Page number from manuscript |
|---|---|---|---|
| **Title and abstract** | 1 | (*a*) Indicate the study's design with a commonly used term in the title or the abstract | 2 |
| | | (*b*) Provide in the abstract an informative and balanced summary of what was done and what was found | 2-3 |
| **Introduction** | | | |
| Background/rationale | 2 | Explain the scientific background and rationale for the investigation being reported | 5-6 |
| Objectives | 3 | State specific objectives, including any prespecified hypotheses | 6 |
| **Methods** | | | |
| Study design | 4 | Present key elements of study design early in the paper | 7 |
| Setting | 5 | Describe the setting, locations, and relevant dates, including periods of recruitment, exposure, follow-up, and data collection | 7-9 |
| Participants | 6 | (*a*) Give the eligibility criteria, and the sources and methods of selection of participants | 7-9 |
| Variables | 7 | Clearly define all outcomes, exposures, predictors, potential confounders, and effect modifiers. Give diagnostic criteria, if applicable | 8-9 |
| Data sources/ measurement | 8* | For each variable of interest, give sources of data and details of methods of assessment (measurement). Describe comparability of assessment methods if there is more than one group | 8-9 |
| Bias | 9 | Describe any efforts to address potential sources of bias | NA |
| Study size | 10 | Explain how the study size was arrived at | 7 |
| Quantitative variables | 11 | Explain how quantitative variables were handled in the analyses. If applicable, describe which groupings were chosen and why | 9-10 |
| Statistical methods | 12 | (*a*) Describe all statistical methods, including those used to control for confounding | 9-10 |
| | | (*b*) Describe any methods used to examine subgroups and interactions | 9-10 |
| | | (*c*) Explain how missing data were addressed | NA |
| | | (*d*) If applicable, describe analytical methods taking account of sampling strategy | NA |
| | | (*e*) Describe any sensitivity analyses | NA |
| **Results** | | | |
| Participants | 13* | (a) Report numbers of individuals at each stage of study—eg numbers potentially eligible, examined for eligibility, confirmed eligible, included in the study, completing follow-up, and analysed | 10 |
| | | (b) Give reasons for non-participation at each stage | 7 |
| | | (c) Consider use of a flow diagram | Figure 1 |
| Descriptive data | 14* | (a) Give characteristics of study participants (eg demographic, clinical, social) and information on exposures and potential | 10-11 |



For peer review only - http://bmjopen.bmj.com/site/about/guidelines.xhtml

For peer review only

| | | | |
|---|---|---|---|
| | | confounders | |
| | | (b) Indicate number of participants with missing data for each variable of interest | 10-11 |
| Outcome data | 15* | Report numbers of outcome events or summary measures | 12-15 |
| Main results | 16 | (*a*) Give unadjusted estimates and, if applicable, confounder-adjusted estimates and their precision (eg, 95% confidence interval). Make clear which confounders were adjusted for and why they were included | 12-15 |
| | | (*b*) Report category boundaries when continuous variables were categorized | NA |
| | | (*c*) If relevant, consider translating estimates of relative risk into absolute risk for a meaningful time period | NA |
| Other analyses | 17 | Report other analyses done—eg analyses of subgroups and interactions, and sensitivity analyses | NA |
| **Discussion** | | | |
| Key results | 18 | Summarise key results with reference to study objectives | 16 |
| Limitations | 19 | Discuss limitations of the study, taking into account sources of potential bias or imprecision. Discuss both direction and magnitude of any potential bias | 19 |
| Interpretation | 20 | Give a cautious overall interpretation of results considering objectives, limitations, multiplicity of analyses, results from similar studies, and other relevant evidence | 16-19 |
| Generalisability | 21 | Discuss the generalisability (external validity) of the study results | 19-20 |
| **Other information** | | | |
| Funding | 22 | Give the source of funding and the role of the funders for the present study and, if applicable, for the original study on which the present article is based | 22 |

*Give information separately for exposed and unexposed groups.

**Note:** An Explanation and Elaboration article discusses each checklist item and gives methodological background and published examples of transparent reporting. The STROBE checklist is best used in conjunction with this article (freely available on the Web sites of PLoS Medicine at http://www.plosmedicine.org/, Annals of Internal Medicine at http://www.annals.org/, and Epidemiology at http://www.epidem.com/). Information on the STROBE Initiative is available at www.strobe-statement.org.

For peer review only



For peer review only - http://bmjopen.bmj.com/site/about/guidelines.xhtml

BMJ Open

# The impact of COVID-19 on household food insecurity and interlinkages with child feeding practices and coping strategies in Uttar Pradesh, India

| | |
|---|---|
| Journal: | *BMJ Open* |
| Manuscript ID | bmjopen-2021-048738.R1 |
| Article Type: | Original research |
| Date Submitted by the Author: | 08-Mar-2021 |
| Complete List of Authors: | Nguyen, Phuong Hong; International Food Policy Research Institute, Poverty, Health and Nutrition Division<br>Kachwaha, Shivani; International Food Policy Research Institute, Poverty, Health and Nutrition Division<br>Pant, Anjali; International Food Policy Research Institute, Poverty, Health and Nutrition Division<br>Tran, Lan; FHI Solutions, Alive &Thrive<br>Ghosh, Sebanti; FHI Solutions, Alive &Thrive<br>Sharma , Praveen ; FHI Solutions, Alive &Thrive<br>Shastri , Vishal ; FHI Solutions, Alive &Thrive<br>Escobar-Alegria , Jessica ; FHI Solutions, Alive &Thrive<br>Avula, Rasmi; International Food Policy Research Institute, Poverty, Health and Nutrition Division<br>Menon, Purnima; International Food Policy Research Institute, Poverty, Health and Nutrition Division |
| <b>Primary Subject Heading</b>: | Global health |
| Secondary Subject Heading: | Nutrition and metabolism, Public health |
| Keywords: | COVID-19, Nutrition < TROPICAL MEDICINE, Public health < INFECTIOUS DISEASES |
| | |

SCHOLARONE™
Manuscripts

*I, the Submitting Author has the right to grant and does grant on behalf of all authors of the Work (as defined in the below author licence), an exclusive licence and/or a non-exclusive licence for contributions from authors who are: i) UK Crown employees; ii) where BMJ has agreed a CC-BY licence shall apply, and/or iii) in accordance with the terms applicable for US Federal Government officers or employees acting as part of their official duties; on a worldwide, perpetual, irrevocable, royalty-free basis to BMJ Publishing Group Ltd ("BMJ") its licensees and where the relevant Journal is co-owned by BMJ to the co-owners of the Journal, to publish the Work in this journal and any other BMJ products and to exploit all rights, as set out in our licence.*

*The Submitting Author accepts and understands that any supply made under these terms is made by BMJ to the Submitting Author unless you are acting as an employee on behalf of your employer or a postgraduate student of an affiliated institution which is paying any applicable article publishing charge ("APC") for Open Access articles. Where the Submitting Author wishes to make the Work available on an Open Access basis (and intends to pay the relevant APC), the terms of reuse of such Open Access shall be governed by a Creative Commons licence – details of these licences and which Creative Commons licence will apply to this Work are set out in our licence referred to above.*

*Other than as permitted in any relevant BMJ Author's Self Archiving Policies, I confirm this Work has not been accepted for publication elsewhere, is not being considered for publication elsewhere and does not duplicate material already published. I confirm all authors consent to publication of this Work and authorise the granting of this licence.*

For peer review only

# The impact of COVID-19 on household food insecurity and interlinkages with child feeding practices and coping strategies in Uttar Pradesh, India

**Authors:**

Phuong Hong Nguyen[1], Shivani Kachwaha[2], Anjali Pant[2], Lan Mai Tran[3], Sebanti Ghosh[4], Praveen Kumar Sharma[4], Vishal Dev Shastri[4], Jessica Escobar-Alegria[5], Rasmi Avula[2], Purnima Menon[2]

**Author affiliations:**

[1]Poverty, Health and Nutrition Division, International Food Policy Research Institute (IFPRI), Washington, DC, USA

[2]Poverty, Health and Nutrition Division, IFPRI, New Delhi, India

[3]Alive &Thrive, FHI Solutions, Hanoi, Vietnam

[4]Alive &Thrive, FHI Solutions, New Delhi, India

[5]Alive &Thrive, FHI Solutions, Washington, DC, USA

***Corresponding author:**

Phuong Hong Nguyen, Poverty, Health and Nutrition Division, International Food Policy Research Institute, 2005 I Street, NW, Washington DC, 20005. Phone: 202-826-4088. Email: P.H.Nguyen@cgiar.org

**Word count: 3481** (from introduction to conclusion, not including tables)

**Abbreviations**

| | |
|---|---|
| AOR: | : Adjusted odds ratio |
| COVID-19 | : Coronavirus |
| HFI | : Household food insecurity |
| PDS | : Public distribution system |
| SES | : Socio-economic status |
| THR | : Take-home rations |



For peer review only - http://bmjopen.bmj.com/site/about/guidelines.xhtml

**ABSTRACT**

**Introduction**: The Coronavirus (COVID-19) pandemic has profound negative impacts on people's lives, but little is known on the effect of COVID-19 on household food insecurity (HFI) in poor setting resources. This study assessed changes in HFI during the pandemic and examined the interlinkages between HFI with child feeding practices and coping strategies in Uttar Pradesh, India.

**Methods**: We conducted a longitudinal quantitative survey with 569 mothers with children <2y in December 2019 (in-person) and August 2020 (by phone). We measured HFI by using the Household Food Insecurity Access Scale and examined the changes in HFI during the pandemic using the Wilcoxon matched-pairs signed-rank tests. We then assessed child feeding practices and coping strategies by HFI status using multivariable regression models, adjusting for child, maternal, and household characteristics.

**Results**: HFI increased sharply from 21% in December 2019 to 80% in August 2020, with 62% households changing the status from food secure to insecure over this period. Children in newly or consistently food-insecure households were less likely to consume a diverse diet (adjusted odds ratio, AOR: 0.57, 95% CI: 0.34, 0.95 and AOR: 0.51, 95% CI: 0.23, 1.12, respectively) compared to those in food-secure households. Households with consistent food insecurity were more likely to engage in coping strategies such as reducing other essential non-food expenditures (AOR: 2.2, 95% CI: 1.09, 4.24), borrowing money to buy food (AOR: 4.3, 95% CI: 2.31, 7.95), or selling jewelry (AOR: 5.0, 95% CI: 1.74, 14.27) to obtain foods. Similar findings were observed for newly food-insecure households.

**Conclusions:** COVID-19 posed a significant risk to HFI which in turn had implications for child feeding practices and coping strategies. Our findings highlight the need for further investment in



For peer review only - http://bmjopen.bmj.com/site/about/guidelines.xhtml

targeted social protection strategies and safety nets as part of multisectoral solutions to improve HFI during and after COVID-19.

**Keywords:** COVID-19, child feeding practices, coping strategies, household food insecurity, India, pandemic

For peer review only



For peer review only - http://bmjopen.bmj.com/site/about/guidelines.xhtml

**Strengths and limitations of this study**

- Using longitudinal surveys with a cohort of mothers with children <2 years, our study provides unique evidence of changes in household food insecurity 6 months after the onset of COVID-19 in the context of a low-middle-income country.
- Our study bridges the gap in literature on the interlinkages between household food insecurity with child feeding practices and coping strategies to obtain food to deal with household economic hardships during the pandemic.
- Our study demonstrates the feasibility of gathering information on household food insecurity via digital data collection methods but indicates some challenges including low response rate and inability to reach some of the poorest or most vulnerable households through phone surveys.
- Since all mothers in our study had children <6 months in December 2019, we were unable to obtain information on complementary feeding to compare child feeding practices before and during COVID pandemic.



For peer review only - http://bmjopen.bmj.com/site/about/guidelines.xhtml

## INTRODUCTION

The Coronavirus disease (COVID-19) has profound and wide-ranging public health impacts and poses a significant global threat to development. Beyond the direct impacts from the virus, the pandemic will likely have a range of indirect consequences on food insecurity, child malnutrition, morbidity and mortality through disruptions in health and nutrition services, food supply chains, and livelihoods.[1-4] Early estimates suggest that potential disruptions of health systems and decreased access to food could lead to 1,157,000 additional child deaths and 56,700 additional maternal deaths.[5] Further, disruptions caused by the pandemic may affect households in multiple other ways including employment and income loss, mobility constraints, and household stress. Experts have warned about the potential consequences of COVID-19, ruining decades of progress, making it unlikely for low and middle income countries to reach the sustainable development goal to "end hunger, achieve food security and improved nutrition and promote sustainable agriculture" by 2030.[6]

There have been growing concerns on the impact of COVID-19 on household food insecurity (HFI).[7 8] Disruptions caused by the pandemic have the potential to influence all "four pillars" of food security including availability, access, utilization, and stability.[9] The pandemic may influence HFI directly on the supply side by disrupting food systems (such as primary food production, stability of food production, processing, food reserve stockpiles, and marketing) as well as indirectly on the demand side due to impact of lockdowns on households' incomes, physical access to food, and economic access to food.[10 11]

The impact of COVID-19 on HFI and poor health outcomes is complex, multilevel, and bidirectional.[4] At the household and individual levels, food insecurity is hypothesized to be a risk factor for both short- and long-term health outcomes through key three pathways: household stress (due to worrying about health issues, job loss and strained finances, and disconnection from social



For peer review only - http://bmjopen.bmj.com/site/about/guidelines.xhtml

support systems), behavioral coping mechanisms (engaging in high-risk behavior, compromising health care activities for foods, poor mental health and inadequate child feeding and nurturing), and inflammatory pathways.[4] Expected negative consequences on food, nutrition, and health security of vulnerable groups including young children, pregnant, and lactating women may further exacerbate existing social and health inequities.[12]

Despite established frameworks and global understanding of the threat to HFI during the pandemic, empirical investigations are very limited to date. Available information on HFI was mainly collected during the pandemic [13-16] and very few studies have examined the dynamic changes of HFI over the COVID pandemic's evolution in low-and middle-income countries (LMICs) [17 18], particularly in the South Asian or Indian context. India is facing a double crisis- COVID-19 and food insecurity,[19] carrying the second highest burden of COVID-19 in the world with nearly 8 million total confirmed cases and 119,502 deaths as of 28th October 2020.[20] Yet only few studies are available on food security using data at the farmer and supply-side level,[21 22] and negligible evidence on the demand side. Very little is known about how women and children within households may be affected by HFI. Further, there is lack of empirical evidence on the changes in HFI during the pandemic. Addressing this knowledge gap is critical for action, specifically at this decisive time in India when the COVID-19 trajectory is still uncertain, and there is concern about potential spikes in the coming months. Our study seeks to address this gap in the current literature with the objectives to 1) assess the changes in HFI before and during the pandemic in Uttar Pradesh, India; and 2) examine the interlinkages between HFI with child feeding practices and coping strategies to deal with household economic hardships and obtain foods.

# METHODS

## Design

This study is a follow-up of a cluster-randomized trial (2017-2019) which aimed to assess the impact of strengthening delivery of maternal nutrition interventions, including micronutrient supplements and intensifying interpersonal counseling and community mobilization, implemented through government ANC services in Uttar Pradesh, India.[23] Details of the parent study have been described elsewhere.[24] Briefly, we conducted in-person repeated cross-sectional surveys of 1,800 recently delivered women as part of the cluster-randomized trial.[23] The end-line data collection was conducted in December 2019, prior to the onset of COVID-19 pandemic, providing an opportunity for a pre-and-post assessment of the effect of COVID-19 on food insecurity in this context.

## Data sources

The household survey was conducted with mothers of children <2 years old following the same study design and sampling frame as in the cluster-randomized trial. Of the 1,849 mothers surveyed at endline from the parent study in December 2019, 587 could be reached for a phone interview in August 2020, yielding a response rate of 32% **(Figure 1).** Reasons for not being able to conduct phone survey included unavailable phone number (n=388), phone unreachable or switched off (n=667), wrong number (n=136), refusal to participate (n=63) and child death (n=9). Reasons for losses to follow-up in the phone survey were similar between intervention and comparison areas (results not shown). The total sample of non-pregnant mothers (n=569) interviewed in both surveys were used for the analysis.

**<Insert Figure 1 here>**



For peer review only - http://bmjopen.bmj.com/site/about/guidelines.xhtml

**Variables**

Household food security was measured before (in-person) and during the pandemic (by phone) using the standard FANTA/USAID's Household Food Insecurity Access Scale.[25] A recent study in Mexico examined the internal validity of food insecurity scales administered through in-person vs. phone surveys and found phone surveys were a feasible strategy to measure food security during COVID-19.[18] Mothers were asked nine questions related to the household's experience of food insecurity in the 30 days preceding the survey. These questions capture 3 main domains of household food insecurity: anxiety and uncertainty about the household food supply (1 item), insufficient quality (3 items), and insufficient quantity and its physical consequences (5 items). We reported the percentage of households that experienced 1) any food insecurity occurrence among nine questions, 2) any of a specific domain, and 3) food insecurity condition categorized as food-secure and mild, moderately, or severely food-insecure.

Information on child feeding practices were assessed using the standard WHO indicators [26], on the basis of the maternal recall of all foods and liquids consumed by the child in different time periods of the previous 24 hours before the survey. All food items were categorized into the 7 food groups used in the WHO guideline:[26] 1) starchy staple foods, 2) legumes and nuts, 3) dairy products (milk, yogurt, and cheese), 4) flesh foods, 5) eggs, 6) vitamin A rich fruits and vegetables, and 7) other fruits and vegetables. Minimum dietary diversity was defined as children who consumed foods from 4 or more out of 7 food groups in the previous 24 hours. Data for complementary feeding practices were not available during the in-person survey in December 2019, because all mothers had children <6 months during that time.

Households were also asked about access to social protection, especially food supplementation they received for mothers and children from the government during the lockdown

For peer review only



For peer review only - http://bmjopen.bmj.com/site/about/guidelines.xhtml

period and during the 30 days prior to the survey, such as take-home rations (THR) and use of public distribution system (PDS). Finally, information on different coping strategies that the household had to engage in the past 30 days due to lack of food was collected, including spending savings, reducing essential non-food expenditure, borrowing money, or selling jewelry/gold.

Other potential factors associated with food security or child feeding practices were obtained for mothers (age, education level, and occupation), child (age and sex), and households (religion, scheduled caste/tribal - designated historically disadvantaged groups in India, number of children <5y, and household socio-economic status- SES). The SES index (collected in person survey) was constructed using a principal component analysis from multiple variables including household ownership of assets, livestock, and housing quality.[27]

**Data analysis**

We compared background characteristics of the analytic sample (mothers who completed both surveys, in-person survey before COVID and phone survey during COVID) and the non-analytic sample (those who completed in-person surveys only) using student t-test (for continuous variables) and chi-square test (for categorical variables). We used descriptive analysis to report HFI before and during the pandemic and child feeding practices. We examined changes in HFI before and during the pandemic using Wilcoxon matched-pairs signed-rank tests.

To examine differences in child feeding practices and coping strategies by food insecurity status, we created three categories of households: 1) food secure (households that were food secure before and during COVID-19 pandemic), 2) consistently food insecure (households that were food insecure before and during COVID-19); 3) newly food insecure (households that were food secure before COVID-19 but became food insecure during the pandemic). We then compared child feeding practices and coping strategies among the three categories using multivariable regression models,



For peer review only - http://bmjopen.bmj.com/site/about/guidelines.xhtml

adjusting for child age and sex, breastfeeding status, mother's age, religion, education, scheduled caste, number of children <5y in the household and household SES. We also examined uptake of social protection programs such as food supplementation and cash transfer as potential strategies to improve HFI. All statistical analyses were undertaken using Stata version 16. Statistical significance was defined as p-value < 0.05.

**Ethical approval**

Informed consent in the local language was obtained from mothers, frontline workers, and block managers prior to their participation in the study. The research protocol received ethical clearance from the Institutional Review Board at the International Food Policy Research Institute (IRB #00007490) and the Suraksha Independent Ethics Committee in India (IRB #2017-10-9094). Additional permissions for data collection were provided by the State Government of Uttar Pradesh.

**Patient and public involvement statement**

Patients or the public were not involved in the design, or conduct, or reporting, or dissemination plans of our research.

## RESULTS

**Characteristics of the study sample**

At the time of in-person survey in December 2019, all mothers had an infant between the ages of 0–5.9 months of age with an average age of 3 months (Table 1). On average, mothers were ~26 years and the majority of them (>90%) were housewives. Nearly all women were Hindu (92%) and nearly half of them belonged to a backward community (44-47%). Mothers in the final analytic sample had higher education (8.2 vs. 6.7 years of schooling, p<0.001) and lived in wealthier (27 vs. 17% in quintile 5, p<0.001) and more food secure households (79 vs. 75%, p=0.08) compared to



For peer review only - http://bmjopen.bmj.com/site/about/guidelines.xhtml

those in the non-analytic sample. Mothers belonging to intervention and control areas of the maternal nutrition intervention (from 2017 to 2019) were equally represented in the analytic sample. In the follow-up phone survey in August 2020- children were on average 11.6 months old (ranging between 8 and 14 months).

**Table 1: Background characteristics[1] of the study sample that participated in surveys before and during the COVID pandemic (December 2019 and August 2020)**

For peer review only

| | Analytic sample (both in person and phone surveys before and during the pandemic) (n= 569) | Non-analytic sample (only in person survey before the pandemic) (n= 1,280) | p |
|---|---|---|---|
| Age of respondent mother (years) | 25.5 (3.8) | 25.7 (4.0) | 0.47 |
| Education (years) | 8.2 (4.3) | 6.7 (4.6) | <0.001 |
| Never attended school | 14.1 | 24.8 | <0.001 |
| Primary school (grade1-5) | 13.9 | 16.3 | |
| Middle school (grade 6-9) | 24.3 | 24.7 | |
| High school (grade 10-12) | 30.1 | 23.3 | |
| Graduate and above | 17.8 | 10.9 | |
| Occupation as housewife | 91.7 | 93.0 | 0.35 |
| Child age, months | 3.0 (1.6) | 2.8 (1.6) | 0.041 |
| Child sex (male) | 49.0 | 49.5 | 0.84 |
| Number of children <5y | 1.6 (0.7) | 1.7 (0.7) | 0.60 |
| Religion as Hindu | 93.7 | 91.1 | 0.061 |
| Caste category | | | |
| Scheduled caste/tribe | 38.3 | 38.4 | 0.25 |
| Other Backward Class | 44.3 | 47.0 | |
| General/others | 17.4 | 14.5 | |
| Household socio-economic status | | | |
| Quintile 1 | 11.6 | 23.8 | <0.001 |
| Quintile 2 | 19.2 | 20.4 | |
| Quintile 3 | 18.1 | 20.9 | |
| Quintile 4 | 24.6 | 18.0 | |
| Quintile 5 | 26.5 | 17.0 | |
| Household food security status | | | |
| Food secure | 79.3 | 74.5 | 0.08 |
| Mildly food insecure | 5.6 | 5.9 | |
| Moderate food insecure | 5.1 | 5.3 | |



For peer review only - http://bmjopen.bmj.com/site/about/guidelines.xhtml

| | | |
|---|---|---|
| Severe food insecure | 10.0 | 14.3 |
| Maternal nutrition (2017-2019) | | |
| Intervention area | 282 | 640 |
| Comparison area | 287 | 640 |

[1]Background data presented in this table were from in -person survey in December 2019.

## Changes in food security status during the COVID pandemic

Prior to the pandemic, 21% of households were identified as food insecure. Six months into the pandemic, the prevalence of any food insecurity increased from 21 to 80%, of which mildly, moderately, and severely food insecure households increased by 14 percentage points (pp), 25 pp and 20 pp, respectively (Figure 2A). Overall, 62% households changed from being food secure to food insecure during the pandemic. HFI experiences sharply increased for each domain. For example, the prevalence of anxiety and uncertainty about the household food supply, insufficient quality of food, and insufficient quantity of food consumed during the pandemic were 45, 78, and 42%, respectively, which was much higher than before the pandemic (12, 18, and 14%, respectively) (Figure 2B).

**<Insert Figure 2 here>**

## Child feeding practices during the COVID pandemic

Child feeding practices are of major concern, with only 19% of children achieving minimum dietary diversity (≥ 4 food groups). An extremely low proportion of children were fed flesh foods (1%), eggs (1%) and vitamin-A rich fruits and vegetables (4%). One-third of the children consumed other fruits and vegetables and nearly two-thirds consumed legumes and nuts in the 24 hours prior to the survey (Figure 3).

**<Insert Figure 3 here>**



For peer review only - http://bmjopen.bmj.com/site/about/guidelines.xhtml

**Association between food insecurity status and child feeding practices during the COVID pandemic**

Children living in households that became food-insecure since the pandemic were less likely to consume a diversified diet (18 vs. 28%; adjusted odds ratio, AOR: 0.57, 95% CI: 0.34, 0.95) as well as legumes and nuts (57 vs. 69%; AOR: 0.61, 95% CI: 0.38, 0.97) compared to children living in consistently food secure households (Table 2).Child feeding practices were worse in the households that were food insecure at both times. Specifically, fewer children in consistently food-insecure households consumed a diverse diet (12.4 vs. 28%; AOR: 0.51, 95% CI: 0.23, 1.12) and other fruits and vegetables (21 vs. 40%; AOR: 0.50, 95% CI: 0.26, 0.97) compared to those in food secure households.

For peer review only

**Table 2: Association between child dietary diversity and household food insecurity status during the pandemic**

| | Currently food secure n=116 | Newly food insecure[1] n=354 | Consistent food insecurity n=99 | New food insecurity vs. food secure[2] | | | | Consistent food insecurity vs. food secure[2] | | | |
|---|---|---|---|---|---|---|---|---|---|---|---|
| | % | % | % | Crude OR (95%CI) | p | Adjusted OR (95%CI) | p | Crude OR (95%CI) | p | Adjusted OR (95%CI) | p |
| Grain | 79.3 | 80.8 | 78.8 | 1.1 (0.65,1.85) | 0.73 | 0.98 (0.57, 1.69) | 0.95 | 0.97 (0.50,1.87) | 0.93 | 0.87 (0.43, 1.77) | 0.64 |
| Legumes and nuts | 69.0 | 56.8 | 55.6 | 0.59 (0.38,0.93) | 0.02 | 0.61 (0.38, 0.97) | 0.04 | 0.56 (0.32,0.98) | 0.04 | 0.69 (0.38, 1.25) | 0.22 |
| Dairy | 74.1 | 76.3 | 79.8 | 1.12 (0.69,1.82) | 0.64 | 1.22 (0.74, 2.01) | 0.43 | 1.38 (0.72,2.62) | 0.33 | 1.72 (0.87, 3.41) | 0.12 |
| Flesh foods | 0.9 | 0.6 | 2.0 | 0.66 (0.06,7.36) | 0.74 | 0.63 (0.05, 7.47) | 0.72 | 2.37 (0.21,26.55) | 0.48 | 1.46 (0.09, 23.2) | 0.79 |
| Eggs | 0.9 | 1.1 | 1.0 | 1.33 (0.15,12.02) | 0.80 | 1.10 (0.11, 10.5) | 0.94 | 1.17 (0.07,19.01) | 0.91 | 0.87 (0.04, 17.0) | 0.93 |
| Vit A rich fruits and vegetables | 4.3 | 4.3 | 2.0 | 0.99 (0.35,2.79) | 0.99 | 0.77 (0.26, 2.26) | 0.64 | 0.46 (0.09,2.41) | 0.36 | 0.31 (0.05, 1.79) | 0.19 |
| Other fruits and vegetables | 39.7 | 33.5 | 21.2 | 0.77 (0.50,1.18) | 0.17 | 0.73 (0.46, 1.16) | 0.18 | 0.41 (0.22,0.75) | 0.004 | 0.50 (0.26, 0.97) | 0.042 |
| Minimum dietary diversity (≥ 4 food groups) | 28.1 | 17.9 | 12.4 | 0.56 (0.34,0.91) | 0.02 | 0.57 (0.34, 0.95) | 0.03 | 0.36 (0.17,0.75) | 0.006 | 0.51 (0.23, 1.12) | 0.09 |

[1]Currently food secure was defined as households who were food secure before and during COVID-19 pandemic and those who were food insecure at some point before but were no longer food insecure during the pandemic, consistent food insecurity was defined as both food insecure before and during COVID-19; newly food insecurity insecure was defined as food secure before COVID-19 but became food insecure during the pandemic. [2]Model controlled for child age, sex, breastfeeding status, mother's age, education, caste, religion, number of children<5y, and household SES.



For peer review only - http://bmjopen.bmj.com/site/about/guidelines.xhtml

**Challenges faced during the pandemic**

The key challenges faced by households in consuming food in the last 7 days preceding the survey included non-availability of funds to buy food (59%), non-availability of foods in market area (21%), increase in food prices (17%), and inability to travel or transport issues (21%). The pandemic-related challenges had resulted in unemployment/loss of income in 78.4% households (**Figure 4**).

**<Insert Figure 4 here>**

**Coping strategies and household food insecurity status during the COVID pandemic**

More than 60% of households disbursed their savings and reduced their expenses on health and non-food essentials to meet food and other requirements, irrespective of their food security status (Table 3). Households experiencing food insecurity were more likely to engage in coping strategies related to obtaining food including reducing their expenditure on non-food essentials (AOR: 1.7, 95% CI: 1.08, 2.78 and AOR: 2.2, 95% CI: 1.09, 4.24 for newly and consistently food-insecure households, respectively), borrowing money to buy food (AOR: 3.6, 95% CI: 2.19, 5.80 and AOR: 4.3, 95% CI: 2.31, 7.95, respectively), and selling jewelry (AOR: 3.0, 95% CI: 1.16, 7.92 and AOR: 5.0, 95% CI: 1.74, 14.27, respectively). Additionally, newly food-insecure households were ~2 times more likely to spend saving or sell households/assets/transport means.

For peer review only



For peer review only - http://bmjopen.bmj.com/site/about/guidelines.xhtml

**Table 3: Association between current coping strategies and household food insecurity status during the pandemic**

| | Currently food secure | Newly food insecure[1] | Consistent food insecurity | New food insecurity vs. food secure[2] | | | | Consistent food insecurity vs. food secure[2] | | | |
|---|---|---|---|---|---|---|---|---|---|---|---|
| | n=116 | n=354 | n=99 | | | | | | | | |
| | % | % | % | Crude OR (95%CI) | p | Adjusted OR (95%CI) | p | Crude OR (95%CI) | p | Adjusted OR (95%CI) | p |
| Spent savings | 83.6 | 91.0 | 89.9 | 1.97 (1.07,3.63) | 0.03 | 2.05 (1.09, 3.88) | 0.027 | 1.74 (0.77,3.95) | 0.18 | 1.73 (0.71, 4.18) | 0.23 |
| Reduced health expenditure | 64.7 | 72.0 | 74.7 | 1.41 (0.90,2.20) | 0.13 | 1.33 (0.84, 2.10) | 0.23 | 1.62 (0.90,2.92) | 0.11 | 1.49 (0.79, 2.80) | 0.22 |
| Reduced other essential non-food expenditures such as education and clothes | 66.4 | 77.4 | 81.8 | 1.73 (1.10,2.74) | 0.02 | 1.73 (1.08, 2.78) | 0.024 | 2.28 (1.20,4.32) | 0.01 | 2.15 (1.09, 4.24) | 0.027 |
| Borrowed money to buy food | 25.0 | 54.8 | 63.6 | 3.64 (2.27,5.82) | <0.001 | 3.57 (2.19, 5.80) | <0.001 | 5.25 (2.92,9.44) | <0.001 | 4.29 (2.31, 7.95) | <0.001 |
| Reduced expenses on agricultural, livestock or fisheries inputs | 23.3 | 33.3 | 35.4 | 1.65 (1.02,2.67) | 0.04 | 1.64 (0.99, 2.72) | 0.055 | 1.80 (0.99,3.27) | 0.05 | 1.78 (0.94, 3.38) | 0.078 |
| Selling jewelry/gold | 4.3 | 13.0 | 21.2 | 3.32 (1.28,8.56) | 0.01 | 3.03 (1.16, 7.92) | 0.024 | 5.98 (2.16,16.53) | 0.001 | 4.98 (1.74, 14.27) | 0.003 |
| Selling household goods or productive assets or means of transport | 19.0 | 29.4 | 27.3 | 1.78 (1.06,2.98) | 0.03 | 1.78 (1.03, 3.07) | 0.038 | 1.6 (0.84,3.04) | 0.15 | 1.64 (0.83, 3.26) | 0.16 |

[1]Currently food secure was defined as households who were food secure before and during COVID-19 pandemic and those who were food insecure at some point before but were no longer food insecure during the pandemic, consistent food insecurity was defined as both food insecure before and during COVID-19; newly food insecure was defined as food secure before COVID-19 but became food insecure during the pandemic. [2]Model controlled for mother's age, education, caste, religion, number of children<5y, and household SES.

For peer review only



For peer review only - http://bmjopen.bmj.com/site/about/guidelines.xhtml

**Social protection before and during the COVID pandemic**

The proportion of households where children received take-home rations (THR) from the ICDS program was similar before and during the pandemic and was slightly higher in food-insecure (~63%) compared to food-secure households (55-59%) (Figure 5).Coverage of PDS rations increased significantly during the pandemic for both food-insecure (61 to 71%) and food-secure households (from 49 to 72%); the increase was smaller among beneficiaries from consistently food-insecure compared to those in food-secure households (9.3 pp vs. 23 pp).

**<Insert Figure 5 here>**

## DISCUSSION

In response to global concerns on the impact of COVID-19 on maternal and child food and nutrition insecurity, our study provides unique evidence of changes in HFI before and during the pandemic and its linkages with child feeding practices as well as coping strategies to obtain foods among food secure and insecure households. We found that HFI increased substantially during the pandemic (60 pp), with a large portion related to insufficient quality (78%) and lower levels related to insufficient quantity (42%). Children living in food-insecure households were less likely to consume a diversified diet, mainly due to less consumption of legumes and nuts, fruits, and vegetables. In order to overcome the challenges during the pandemic, households were compelled to engage in several coping strategies related to spending existing savings, reducing household expenditures, selling assets, or borrowing money.

Our findings were consistent with the global literature on the increase in HFI during the pandemic.[28-32] However, most previous studies mainly obtained information during the pandemic and did not have data prior to the onset of the pandemic. A rapid assessment conducted in LMICs including Kenya, Nigeria, Mozambique, and Rwanda showed that 79-87% of



For peer review only - http://bmjopen.bmj.com/site/about/guidelines.xhtml

respondents were worried about lack of sufficient food during COVID-19.[13] Similarly, nearly 90% of households in rural and urban Bangladesh experienced different levels of food insecurity and engaged in financial or food compromised coping strategies.[15] The prevalence of moderate to severe HFI during the COVID-19 lockdown was lower in Peru, affecting 23% of households, with predictors being low income pre-pandemic, income reduction, or running out of savings during the pandemic.[14] Among the few studies with information before and during COVID-19 time, two were from the US, one found 32% increase in HFI since COVID-19 [16] while the other found an increase of 20%.[28] Only two other studies provided estimates of HFI before and during COVID where one found an increase of 14 pp (from 61.1 to 75.1%) in any HFI in Mexico[18] and the other observed an increase of 43.4 pp (from 8.3 to 51.7%) in moderate and severe HFI in Bangladesh.[17] Our study showed much higher magnitude of increase in HFI (~60 pp) compared to other studies, which is a worrisome finding given the high pre-existing levels of food insecurity in India. We also found that HFI was predominantly due to insufficient food quality concerns which was aligned with a previous study which showed increased consumption of high-calorie snack foods and sweets,[28 30] or cheaper highly processed foods.[4]

Our findings indicate challenges to several food security dimensions, including livelihood and income loss, economic and physical access, availability, and utilization. A study on livelihood and dietary effects of COVID-19 with vegetable producers in four states of India reported negative impacts on production, sales, prices, and incomes among majority of farmers,[21] Farm households also reported disruptions to their diets with reduced ability to access nutrient-dense foods, particularly fruit and animal source foods.[21] Another study in Maharashtra, India found disruptions in the urban-rural food supply chain due to the closure of wholesale markets with uncertainties in food supply, declines in market availability, and increase in food prices.[22]



For peer review only - http://bmjopen.bmj.com/site/about/guidelines.xhtml

These findings are complementary to our study and the supply-side insights possibly explain some of the trends we observe in food security, child feeding, and coping strategies.

To our knowledge, infant and child feeding practices during the pandemic have not been explored in the literature. Our findings showed that the diets of children were suboptimal, with only 19% achieving minimum dietary diversity – a similar result compared to a previous study in Uttar Pradesh, India before COVID pandemic (17%).[33] We also found that children living in food-insecure households had much poorer diets than those in food-secure household, but the proportion of children consuming flesh foods, eggs, and vitamin A fruits and vegetables is very low, irrespective of food security status. During the COVID-19 pandemic, child feeding practices have been reported to change, particularly among food-insecure households, due to higher levels of stress, fewer resources, and less access to food and affordability, leading to restrict the quantity and quality of food their children eat and more parents' controlling feeding behaviors.[28] Other studies also showed that mothers of the children in food-insecure households often prioritized shelf-stable foods to deal with food supply disruptions and social-distancing policies, and have a tendency to rely on energy-dense foods for a longer period of time.[4]

We found that all households in our study engaged in some coping strategies to obtain food regardless of HFI status, but food-insecure households were more likely to engage in several such practices. Our findings are consistent with literature stating that the main strategies food-insecure households generally rely on to maintain access to food include shifting within their own spending patterns to prioritize food (reducing expenses on health, other non-food expenditures, or agricultural, livestock or fisheries inputs), relying on social network, or access to government nutrition programs.[4 16] However, all these strategies can easily be impacted when COVID pandemic severely affects the entire household budget, or social-distancing policies


For peer review only - http://bmjopen.bmj.com/site/about/guidelines.xhtml

could affect network access. The coping strategies households have adopted to obtain food during COVID-19 will run out and will not suffice for preventing HFI from getting worse if the pandemic crisis continues.

Social protection strategies are an important intervention to address the rising levels of HFI in the context of COVID-19, particularly for low-income countries.[34] A global review of evidence by the World Bank found that India increased coverage of cash transfers from ~2% before the pandemic to about 15% during COVID-19.[35] The Indian government also initiated home-delivery of take-home rations for pregnant and lactating women and children and provided one-month free supply of wheat and rice to the poorest ration card holders through the public distribution system.[36] Our findings on the increased access to PDS during COVID-19 align with previous conclusions about the important role of the program as an essential component of the Government's response to food insecurity.[37] Despite these measures, food supplementation was received among just over half of households and the increase in access to PDS was smaller among beneficiaries that are consistently food insecure compared to the food secure. These results highlight an important opportunity to strengthen the Government's response to reduce food insecurity during and after COVID-19 in the short term by improving efficiency of existing social protection strategies and targeting to the most vulnerable populations.[37,38] A recent costing study conducted in Mexico found it would cost less than 0.06% of the Gross Domestic Product to effectively safeguard families with young children through a cash transfer and basic services subsidy.[39] Other strategies which may be considered include outlining specific recommendations to ensure food security for poor and vulnerable populations as done for other developing countries in Africa[40] and include special initiatives for migrant populations.[41] Certain agricultural reforms may also be considered[42] such as home gardening,[43] diversification of production, and



For peer review only - http://bmjopen.bmj.com/site/about/guidelines.xhtml

strong local market chains[44] to alleviate HFI, improve diets, and reduce reliance on coping strategies due to food insecurity.

Our study followed the cohort of mothers before the pandemic and 6 months after the onset of COVID-19, thus offering a unique and timely contribution to the literature on the magnitude and nature of increase in HFI before and during the pandemic, and its implications for child feeding practices and coping strategies in the context of LMICs with prevailing high HFI. Given the restrictions on movement and contacting people, we were able to mobilize the phone survey to reach mothers and use the same instrument to measure food security over time. Our experience demonstrated the feasibility of gathering information on HFI via digital data collection methods but indicated potential challenges and bias in the background characteristics of respondents interviewed through in-person vs. phone surveys. Mothers who responded to phone survey had slightly higher education and SES background compared to those in the non-analytic sample (only in-person survey), indicating that we may not be able to reach some of the poorest or most vulnerable households through phone surveys. We also experienced similar challenges as other phone surveys[45] including low response rate, several calling schedules during the survey, and potential unknown response bias. Finally, since all mothers in our study had children <6 months in December 2019, we were unable to obtain information on complementary feeding to compare child feeding practices before and during COVID time.

## CONCLUSION

COVID-19 had a significant negative impact on HFI in this context, which in turn had implications for child feeding practices and reliance on coping strategies to obtain foods. Our study highlighted the opportunity to reduce HFI in the short-term with existing resources by



For peer review only - http://bmjopen.bmj.com/site/about/guidelines.xhtml

improving the targeting of social protection benefits to effectively reach the food insecure and make quality diets accessible. Given the great concerns about the expected increase in HFI as the pandemic continues, strengthened multisectoral responses are needed to ensure effective re-establishment of health and nutrition services, food supply chains, and restoration of livelihoods to improve household food security during and after the pandemic.

For peer review only



For peer review only - http://bmjopen.bmj.com/site/about/guidelines.xhtml

## FIGURE LEGENDS

**Figure 1.** Participant flow

**Figure 2.** Food insecurity experienced by mothers and their household members in the previous 30 days before and during the COVID pandemic

**Figure 3:** Child feeding practices during the COVID pandemic

**Figure 4:** The key challenges faced by households during the COVID pandemic

**Figure 5:** Household receipt of social protection benefits before and during the pandemic, by household food insecurity status

For peer review only

## AUTHOR CONTRIBUTIONS

PHN: conceived paper, analysis, drafted manuscript, consolidated comments from all co-authors, revised and finalized paper.

SK: Field work coordination, literature review, drafted some parts of the manuscript, revised, and finalized paper.

AP: Field work coordination, data analyses, drafted some parts of the manuscript, reviewed manuscript.

LMT: data analyses, visualization for data presentation, reviewed the manuscript.

SG, PKS, VDS, JE: data interpretation and its implications, reviewed and edited the manuscript.

RA and PM reviewed the statistical analyses, supported data interpretation, reviewed, and edited the manuscript.

All authors read and approved the final submitted manuscript.

## SOURCE OF FUNDING

Bill & Melinda Gates Foundation through POSHAN, led by International Food Policy Research Institute. Grant number: OPP50838.

## CONFLICT OF INTEREST STATEMENT

Authors declare no conflicts of interest.

## DATA AVAILABILITY STATEMENT

All data relevant to the study are included in the article or uploaded as supplementary information.

For peer review only



For peer review only - http://bmjopen.bmj.com/site/about/guidelines.xhtml

## REFERENCES

1. Tesfaye A, Habte Y, Minten B. COVID-19 is shifting consumption and disrupting dairy value chains in Ethiopia. IFPRI book chapters, in: COVID-19 and global food security, chapter 9, pages 42-45, International Food Policy Research Institute (IFPRI). 2020
2. Hirvonen K, De Brauw A, Abate G. Food Consumption and Food Security during the COVID-19 Pandemic in Addis Ababa. IFPRI Discussion Paper 01964. 2020
3. Headey D, Heidkamp R, Osendarp S, et al. Impacts of COVID-19 on childhood malnutrition and nutrition-related mortality. *Lancet* 2020;396(10250):519-21. doi: 10.1016/S0140-6736(20)31647-0 [published Online First: 2020/07/31]
4. Leddy AM, Weiser SD, Palar K, et al. A conceptual model for understanding the rapid COVID-19-related increase in food insecurity and its impact on health and healthcare. *Am J Clin Nutr* 2020 doi: 10.1093/ajcn/nqaa226 [published Online First: 2020/08/09]
5. Roberton T, Carter ED, Chou VB, et al. Early estimates of the indirect effects of the COVID-19 pandemic on maternal and child mortality in low-income and middle-income countries: a modelling study. *Lancet Glob Health* 2020;8(7):e901-e08. doi: 10.1016/S2214-109X(20)30229-1 [published Online First: 2020/05/15]
6. United Nations. Resolution adopted by the General Assembly on 25 September 2015, Transforming our world: the 2030 Agenda for Sustainable Development (A/RES/70/1) 2015
7. Paslakis G, Dimitropoulos G, Katzman DK. A call to action to address COVID-19-induced global food insecurity to prevent hunger, malnutrition, and eating pathology. *Nutr Rev* 2020 doi: 10.1093/nutrit/nuaa069 [published Online First: 2020/07/12]
8. Cable J, Jaykus LA, Hoelzer K, et al. The impact of COVID-19 on food systems, safety, and security-a symposium report. *Ann N Y Acad Sci* 2020 doi: 10.1111/nyas.14482 [published Online First: 2020/08/30]
9. Laborde D, Martin W, Swinnen J, et al. COVID-19 risks to global food security. *Science* 2020;369(6503):500-02. doi: 10.1126/science.abc4765 [published Online First: 2020/08/01]
10. Devereux S, Bene C, Hoddinott J. Conceptualising COVID-19's impacts on household food security. *Food Secur* 2020:1-4. doi: 10.1007/s12571-020-01085-0 [published Online First: 2020/08/25]
11. Savary S, Akter S, Almekinders C, et al. Mapping disruption and resilience mechanisms in food systems. *Food Secur* 2020:1-23. doi: 10.1007/s12571-020-01093-0 [published Online First: 2020/08/25]
12. Perez-Escamilla R, Cunningham K, Moran VH. COVID-19 and maternal and child food and nutrition insecurity: a complex syndemic. *Matern Child Nutr* 2020;16(3):e13036. doi: 10.1111/mcn.13036 [published Online First: 2020/05/28]
13. GAIN. Impact of COVID-19 on Food Systems: A Situation Report, Edition 3. May 13, 2020. 2020
14. Cañari-Casaño JL, Elorreaga OA, Cochachin-Henostroza O, et al. Social predictors of food insecurity during the stay-at-home order due to the COVID-19 pandemic in Peru. Results from a cross-sectional web-based survey. *medRxiv 2021 Feb 8;2021020621251221 doi: 101101/2021020621251221 Preprint* 2021
15. Das S, Rasul MG, Hossain MS, et al. Acute food insecurity and short-term coping strategies of urban and rural households of Bangladesh during the lockdown period of COVID-19



For peer review only - http://bmjopen.bmj.com/site/about/guidelines.xhtml

pandemic of 2020: report of a cross-sectional survey. *BMJ Open* 2020;10(12):e043365. doi: 10.1136/bmjopen-2020-043365 [published Online First: 2020/12/15]
16. Niles MT, Bertmann F, Belarmino EH, et al. The Early Food Insecurity Impacts of COVID-19. *Nutrients* 2020;12(7) doi: 10.3390/nu12072096 [published Online First: 2020/07/19]
17. Hamadani JD, Hasan MI, Baldi AJ, et al. Immediate impact of stay-at-home orders to control COVID-19 transmission on socioeconomic conditions, food insecurity, mental health, and intimate partner violence in Bangladeshi women and their families: an interrupted time series. *Lancet Glob Health* 2020;8(11):e1380-e89. doi: 10.1016/S2214-109X(20)30366-1 [published Online First: 2020/08/29]
18. Gaitan-Rossi P, Vilar-Compte M, Teruel G, et al. Food insecurity measurement and prevalence estimates during the COVID-19 pandemic in a repeated cross-sectional survey in Mexico. *Public Health Nutr* 2021;24(3):412-21. doi: 10.1017/S1368980020004000 [published Online First: 2020/10/15]
19. Mishra K, Rampal J. The COVID-19 pandemic and food insecurity: A viewpoint on India. *World Development* *https://doiorg/101016/jworlddev2020105068* 2020;135:1-3.
20. Ministry of Health and Family Welfare. COVID-19 INDIA as on : 14 October 2020, 08:00 IST (GMT+5:30) New Delhi, India: Government of India; 2020 [Available from: https://www.mohfw.gov.in/index1.php?lang=1level=1sublinkid=6471lid=4270 accessed 15th October 2020.
21. Harris J, Depenbusch L, Pal AA, et al. Food system disruption: initial livelihood and dietary effects of COVID-19 on vegetable producers in India. *Food Secur* 2020:1-11. doi: 10.1007/s12571-020-01064-5
22. Sukhwani V, Deshkar S, Shaw R. COVID-19 Lockdown, Food Systems and Urban-Rural Partnership: Case of Nagpur, India. *Int J Environ Res Public Health* 2020;17(16) doi: 10.3390/ijerph17165710 [published Online First: 2020/08/14]
23. Nguyen P, Kachwaha S, Tran LM, et al. Strengthening Nutrition Interventions in Antenatal Care Services Had Modest Impacts on Diet Diversity, Micronutrient Intake, and Breastfeeding in Uttar Pradesh, India. *Current Developments in Nutrition,* 2020;4(Supplement_2):1050, https://doi.org/10.93/cdn/nzaa054_122.
24. Nguyen PH, Kachwaha S, Tran LM, et al. Integrating Maternal Nutrition Interventions in Existing Reproductive, Maternal, Newborn and Child Health Services in Uttar Pradesh, India. Alive & Thrive Endline Survey Report. Washington, DC: Alive & Thrive. 2020
25. Coates J, Swindale A, Bilinsky P. Household Food Insecurity Access Scale (HFIAS) for Measurement of Household Food Access: Indicator Guide (v. 3). Washington, D.C.: Food and Nutrition Technical Assistance Project, Academy for Educational Development. 2007
26. WHO. Indicators for assessing infant and young child feeding practices. Part 2: Measurements. http://whqlibdoc.who.int/publications/2010/9789241599290_eng.pdf. World Health Organization: Geneva, 2008.
27. Filmer D, Pritchett LH. Estimating wealth effects without expenditure data--or tears: an application to educational enrollments in states of India. *Demography* 2001;38(1):115-32. [published Online First: 2001/03/03]
28. Adams EL, Caccavale LJ, Smith D, et al. Food insecurity, the home food environment, and parent feeding practices in the era of COVID-19. *Obesity (Silver Spring)* 2020 doi: 10.1002/oby.22996 [published Online First: 2020/08/08]

For peer review only

29. Bene C. Resilience of local food systems and links to food security - A review of some important concepts in the context of COVID-19 and other shocks. *Food Secur* 2020:1-18. doi: 10.1007/s12571-020-01076-1 [published Online First: 2020/08/25]
30. Carroll N, Sadowski A, Laila A, et al. The Impact of COVID-19 on Health Behavior, Stress, Financial and Food Security among Middle to High Income Canadian Families with Young Children. *Nutrients* 2020;12(8) doi: 10.3390/nu12082352 [published Online First: 2020/08/14]
31. Kent K, Murray S, Penrose B, et al. Prevalence and Socio-Demographic Predictors of Food Insecurity in Australia during the COVID-19 Pandemic. *Nutrients* 2020;12(9) doi: 10.3390/nu12092682 [published Online First: 2020/09/06]
32. Owens MR, Brito-Silva F, Kirkland T, et al. Prevalence and Social Determinants of Food Insecurity among College Students during the COVID-19 Pandemic. *Nutrients* 2020;12(9) doi: 10.3390/nu12092515 [published Online First: 2020/08/23]
33. MoHFW. Ministry of Health and Family Welfare (MoHFW), Government of India, UNICEF and Population Council. Comprehensive National Nutrition Survey (CNNS) National Report. New Delhi, India, 2019.
34. Pereira M, Oliveira AM. Poverty and food insecurity can increase as threats of COVID-19 spreads. *Public Health Nutr* 2020:1-10. doi: 10.1017/S1368980020003493 [published Online First: 2020/09/09]
35. Gentilini U, Almenfi M, Orton I, et al. Social Protection and Jobs Responses to COVID-19 : A Real-Time Review of Country Measures. Washington DC: World Bank, 2020.
36. International Food Policy Research Institute. POSHAN COVID-19 Resources: Online archive for COVID-19 related resources on nutrition, early childhood development and food security New Delhi, India: POSHAN; 2020 [Available from: https://poshancovid19.in/Resources.html accessed 15th October 2020.
37. George NA, McKay FH. The Public Distribution System and Food Security in India. *Int J Environ Res Public Health* 2019;16(17) doi: 10.3390/ijerph16173221 [published Online First: 2019/09/06]
38. FAO, IFAD, UNICEF, et al. The State of Food Security and Nutrition in the World 2020. Transforming food systems for affordable healthy diets. Rome, FAO. DOI: https://doi.org/10.4060/ca9692en. 2020
39. Vilar-Compte M, Perez V, Teruel G, et al. Costing of actions to safeguard vulnerable Mexican households with young children from the consequences of COVID-19 social distancing measures. *Int J Equity Health* 2020;19(1):70. doi: 10.1186/s12939-020-01187-3 [published Online First: 2020/05/21]
40. Lawson-Lartego L, Cohen MJ. 10 recommendations for African governments to ensure food security for poor and vulnerable populations during COVID-19. *Food Secur* 2020;12(4):899-902. doi: 10.1007/s12571-020-01062-7 [published Online First: 2020/08/26]
41. Smith MD, Wesselbaum D. COVID-19, Food Insecurity, and Migration. *J Nutr* 2020 doi: 10.1093/jn/nxaa270 [published Online First: 2020/08/26]
42. Kumar A, Padhee AK, Kumar S. How Indian agriculture should change after COVID-19. *Food Secur* 2020:1-4. doi: 10.1007/s12571-020-01063-6 [published Online First: 2020/08/25]



For peer review only - http://bmjopen.bmj.com/site/about/guidelines.xhtml

43. Lal R. Home gardening and urban agriculture for advancing food and nutritional security in response to the COVID-19 pandemic. *Food Secur* 2020:1-6. doi: 10.1007/s12571-020-01058-3 [published Online First: 2020/08/25]
44. Heck S, Campos H, Barker I, et al. Resilient agri-food systems for nutrition amidst COVID-19: evidence and lessons from food-based approaches to overcome micronutrient deficiency and rebuild livelihoods after crises. *Food Security* *https://doiorg/101007/s12571-020-01067-2* 2020
45. Kempf AM, Remington PL. New challenges for telephone survey research in the twenty-first century. *Annu Rev Public Health* 2007;28:113-26. doi: 10.1146/annurev.publhealth.28.021406.144059 [published Online First: 2006/11/11]

For peer review only



For peer review only - http://bmjopen.bmj.com/site/about/guidelines.xhtml

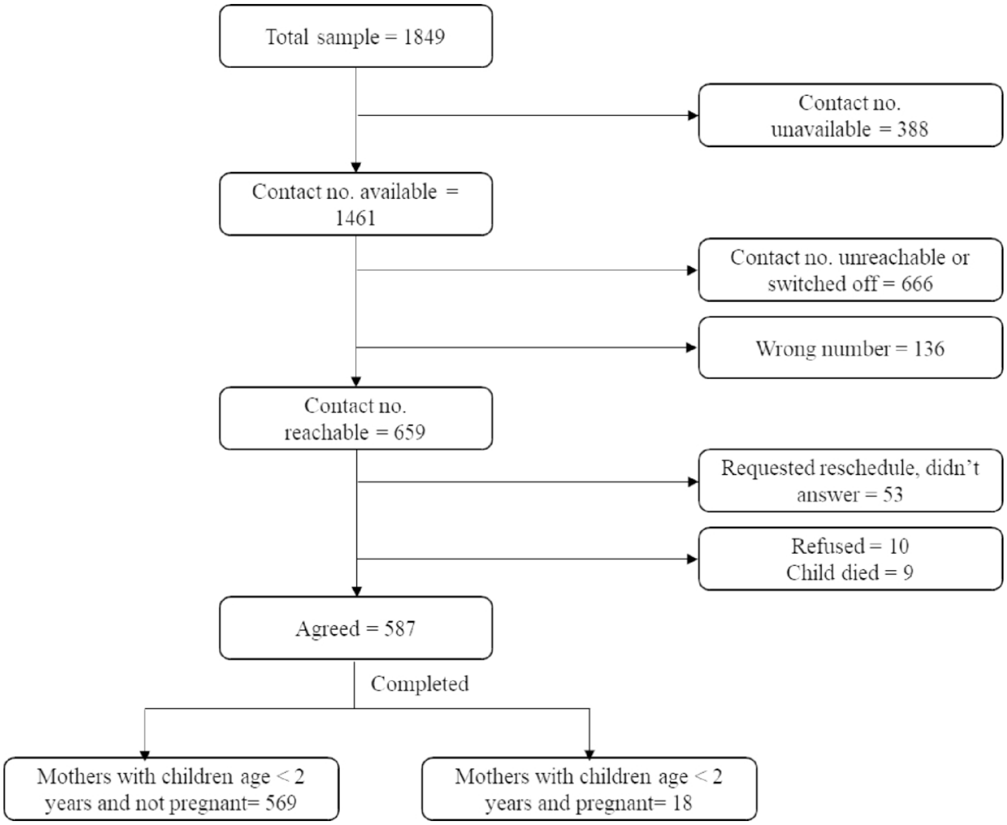


Figure 1. Participant flow

172x140mm (300 x 300 DPI)

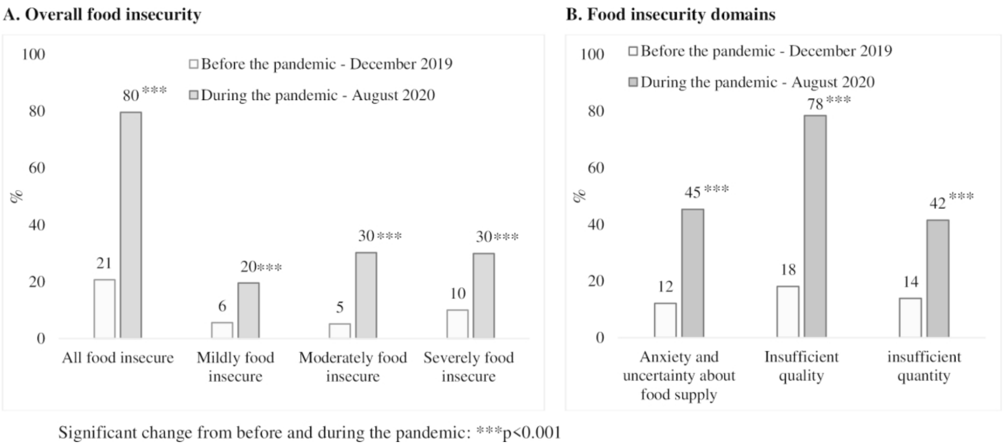


Figure 2. Food insecurity experienced by mothers and their household members in the previous 30 days before and during the COVID pandemic

190x82mm (300 x 300 DPI)

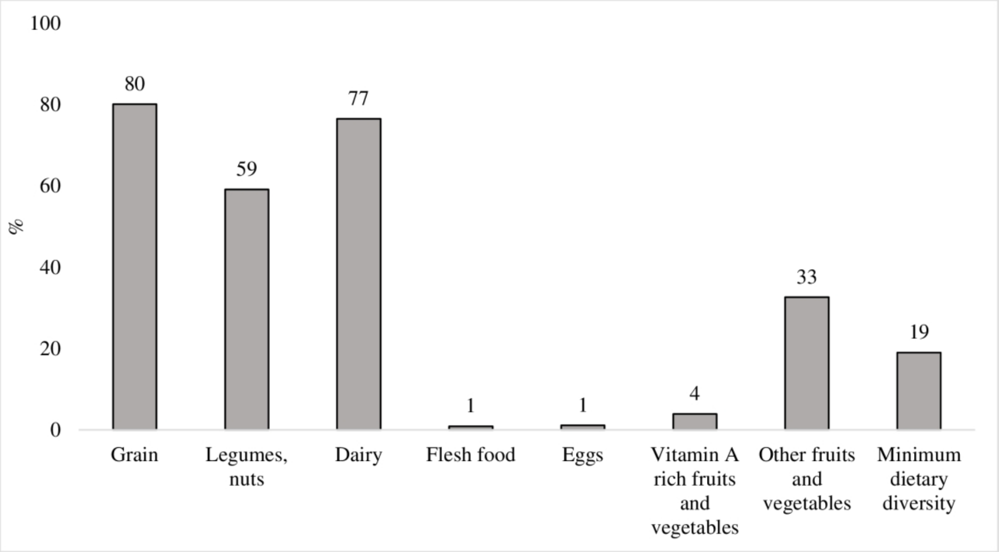


Figure 3: Child feeding practices during the COVID pandemic

168x92mm (300 x 300 DPI)

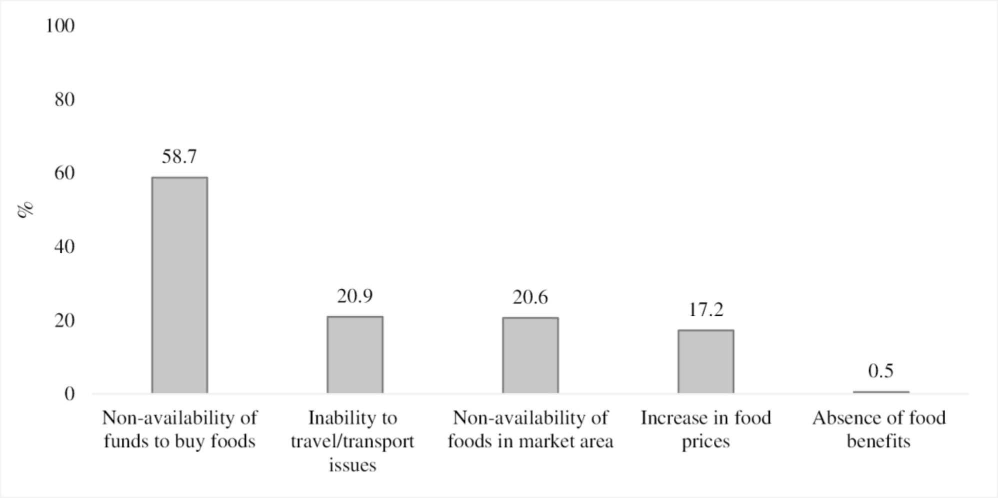


Figure 4: The key challenges faced by households during the COVID pandemic

156x77mm (300 x 300 DPI)

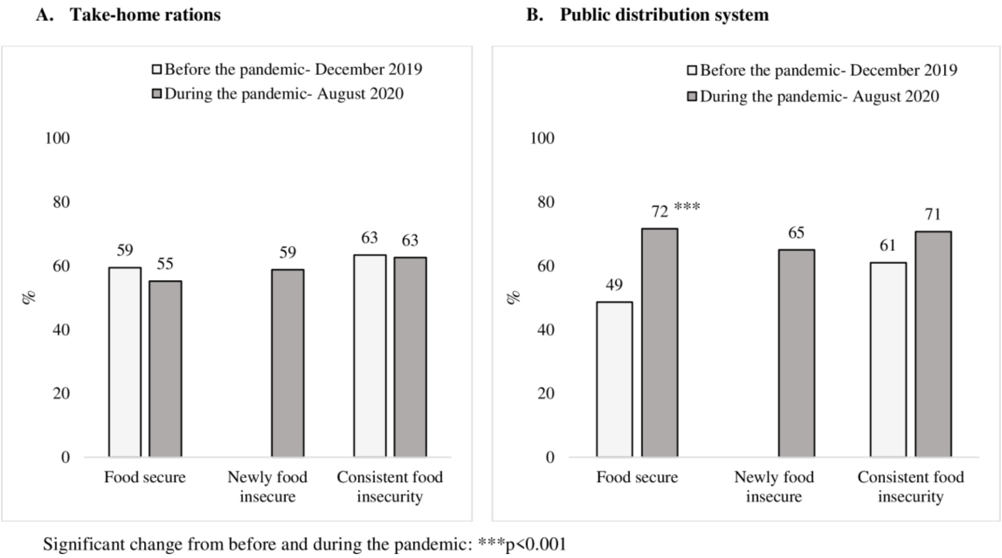


Figure 5: Household receipt of social protection benefits before and during the pandemic, by household food insecurity status

187x102mm (300 x 300 DPI)

## STROBE Statement—Checklist of items that should be included in reports of cross-sectional studies

| | Item No | Recommendation | Page number from manuscript |
|---|---|---|---|
| **Title and abstract** | 1 | (*a*) Indicate the study's design with a commonly used term in the title or the abstract | 2 |
| | | (*b*) Provide in the abstract an informative and balanced summary of what was done and what was found | 2-3 |
| **Introduction** | | | |
| Background/rationale | 2 | Explain the scientific background and rationale for the investigation being reported | 5-6 |
| Objectives | 3 | State specific objectives, including any prespecified hypotheses | 6 |
| **Methods** | | | |
| Study design | 4 | Present key elements of study design early in the paper | 7 |
| Setting | 5 | Describe the setting, locations, and relevant dates, including periods of recruitment, exposure, follow-up, and data collection | 7-9 |
| Participants | 6 | (*a*) Give the eligibility criteria, and the sources and methods of selection of participants | 7-9 |
| Variables | 7 | Clearly define all outcomes, exposures, predictors, potential confounders, and effect modifiers. Give diagnostic criteria, if applicable | 8-9 |
| Data sources/ measurement | 8* | For each variable of interest, give sources of data and details of methods of assessment (measurement). Describe comparability of assessment methods if there is more than one group | 8-9 |
| Bias | 9 | Describe any efforts to address potential sources of bias | NA |
| Study size | 10 | Explain how the study size was arrived at | 7 |
| Quantitative variables | 11 | Explain how quantitative variables were handled in the analyses. If applicable, describe which groupings were chosen and why | 9-10 |
| Statistical methods | 12 | (*a*) Describe all statistical methods, including those used to control for confounding | 9-10 |
| | | (*b*) Describe any methods used to examine subgroups and interactions | 9-10 |
| | | (*c*) Explain how missing data were addressed | NA |
| | | (*d*) If applicable, describe analytical methods taking account of sampling strategy | NA |
| | | (*e*) Describe any sensitivity analyses | NA |
| **Results** | | | |
| Participants | 13* | (a) Report numbers of individuals at each stage of study—eg numbers potentially eligible, examined for eligibility, confirmed eligible, included in the study, completing follow-up, and analysed | 10 |
| | | (b) Give reasons for non-participation at each stage | 7 |
| | | (c) Consider use of a flow diagram | Figure 1 |
| Descriptive data | 14* | (a) Give characteristics of study participants (eg demographic, clinical, social) and information on exposures and potential | 10-11 |



For peer review only - http://bmjopen.bmj.com/site/about/guidelines.xhtml

| | | | |
|---|---|---|---|
| | | confounders | |
| | | (b) Indicate number of participants with missing data for each variable of interest | 10-11 |
| Outcome data | 15* | Report numbers of outcome events or summary measures | 12-15 |
| Main results | 16 | (*a*) Give unadjusted estimates and, if applicable, confounder-adjusted estimates and their precision (eg, 95% confidence interval). Make clear which confounders were adjusted for and why they were included | 12-15 |
| | | (*b*) Report category boundaries when continuous variables were categorized | NA |
| | | (*c*) If relevant, consider translating estimates of relative risk into absolute risk for a meaningful time period | NA |
| Other analyses | 17 | Report other analyses done—eg analyses of subgroups and interactions, and sensitivity analyses | NA |
| **Discussion** | | | |
| Key results | 18 | Summarise key results with reference to study objectives | 16 |
| Limitations | 19 | Discuss limitations of the study, taking into account sources of potential bias or imprecision. Discuss both direction and magnitude of any potential bias | 19 |
| Interpretation | 20 | Give a cautious overall interpretation of results considering objectives, limitations, multiplicity of analyses, results from similar studies, and other relevant evidence | 16-19 |
| Generalisability | 21 | Discuss the generalisability (external validity) of the study results | 19-20 |
| **Other information** | | | |
| Funding | 22 | Give the source of funding and the role of the funders for the present study and, if applicable, for the original study on which the present article is based | 22 |

*Give information separately for exposed and unexposed groups.

**Note:** An Explanation and Elaboration article discusses each checklist item and gives methodological background and published examples of transparent reporting. The STROBE checklist is best used in conjunction with this article (freely available on the Web sites of PLoS Medicine at http://www.plosmedicine.org/, Annals of Internal Medicine at http://www.annals.org/, and Epidemiology at http://www.epidem.com/). Information on the STROBE Initiative is available at www.strobe-statement.org.

For peer review only

BMJ Open

# The impact of COVID-19 on household food insecurity and interlinkages with child feeding practices and coping strategies in Uttar Pradesh, India: A longitudinal community-based study

| | |
|---|---|
| Journal: | *BMJ Open* |
| Manuscript ID | bmjopen-2021-048738.R2 |
| Article Type: | Original research |
| Date Submitted by the Author: | 03-Apr-2021 |
| Complete List of Authors: | Nguyen, Phuong Hong; International Food Policy Research Institute, Poverty, Health and Nutrition Division<br>Kachwaha, Shivani; International Food Policy Research Institute, Poverty, Health and Nutrition Division<br>Pant, Anjali; International Food Policy Research Institute, Poverty, Health and Nutrition Division<br>Tran, Lan; FHI Solutions, Alive &Thrive<br>Ghosh, Sebanti; FHI Solutions, Alive &Thrive<br>Sharma , Praveen ; FHI Solutions, Alive &Thrive<br>Shastri , Vishal ; FHI Solutions, Alive &Thrive<br>Escobar-Alegria , Jessica ; FHI Solutions, Alive &Thrive<br>Avula, Rasmi; International Food Policy Research Institute, Poverty, Health and Nutrition Division<br>Menon, Purnima; International Food Policy Research Institute, Poverty, Health and Nutrition Division |
| <b>Primary Subject Heading</b>: | Global health |
| Secondary Subject Heading: | Nutrition and metabolism, Public health |
| Keywords: | COVID-19, Nutrition < TROPICAL MEDICINE, Public health < INFECTIOUS DISEASES |
| | |

SCHOLARONE™ Manuscripts

*I, the Submitting Author has the right to grant and does grant on behalf of all authors of the Work (as defined in the below author licence), an exclusive licence and/or a non-exclusive licence for contributions from authors who are: i) UK Crown employees; ii) where BMJ has agreed a CC-BY licence shall apply, and/or iii) in accordance with the terms applicable for US Federal Government officers or employees acting as part of their official duties; on a worldwide, perpetual, irrevocable, royalty-free basis to BMJ Publishing Group Ltd ("BMJ") its licensees and where the relevant Journal is co-owned by BMJ to the co-owners of the Journal, to publish the Work in this journal and any other BMJ products and to exploit all rights, as set out in our licence.*

*The Submitting Author accepts and understands that any supply made under these terms is made by BMJ to the Submitting Author unless you are acting as an employee on behalf of your employer or a postgraduate student of an affiliated institution which is paying any applicable article publishing charge ("APC") for Open Access articles. Where the Submitting Author wishes to make the Work available on an Open Access basis (and intends to pay the relevant APC), the terms of reuse of such Open Access shall be governed by a Creative Commons licence – details of these licences and which Creative Commons licence will apply to this Work are set out in our licence referred to above.*

*Other than as permitted in any relevant BMJ Author's Self Archiving Policies, I confirm this Work has not been accepted for publication elsewhere, is not being considered for publication elsewhere and does not duplicate material already published. I confirm all authors consent to publication of this Work and authorise the granting of this licence.*

For peer review only

# The impact of COVID-19 on household food insecurity and interlinkages with child feeding practices and coping strategies in Uttar Pradesh, India: A longitudinal community-based study

**Authors:**

Phuong Hong Nguyen[1], Shivani Kachwaha[2], Anjali Pant[2], Lan Mai Tran[3], Sebanti Ghosh[4], Praveen Kumar Sharma[4], Vishal Dev Shastri[4], Jessica Escobar-Alegria[5], Rasmi Avula[2], Purnima Menon[2]

**Author affiliations:**

[1]Poverty, Health and Nutrition Division, International Food Policy Research Institute (IFPRI), Washington, DC, USA

[2]Poverty, Health and Nutrition Division, IFPRI, New Delhi, India

[3]Alive &Thrive, FHI Solutions, Hanoi, Vietnam

[4]Alive &Thrive, FHI Solutions, New Delhi, India

[5]Alive &Thrive, FHI Solutions, Washington, DC, USA

***Corresponding author:**

Phuong Hong Nguyen, Poverty, Health and Nutrition Division, International Food Policy Research Institute, 2005 I Street, NW, Washington DC, 20005. Phone: 202-826-4088. Email: P.H.Nguyen@cgiar.org

**Word count: 3950** (from introduction to conclusion, not including tables)

**Abbreviations**

AOR: : Adjusted odds ratio
COVID-19 : Coronavirus
HFI : Household food insecurity
PDS : Public distribution system
SES : Socio-economic status
THR : Take-home rations



For peer review only - http://bmjopen.bmj.com/site/about/guidelines.xhtml

## ABSTRACT

**Objectives**: The Coronavirus (COVID-19) pandemic has profound negative impacts on people's lives, but little is known on its effect on household food insecurity (HFI) in poor setting resources. This study assessed changes in HFI during the pandemic and examined the interlinkages between HFI with child feeding practices and coping strategies.

**Design:** A longitudinal survey in December 2019 (in-person) and August 2020 (by phone).

**Setting:** Community-based individuals from 26 blocks in 2 districts in Uttar Pradesh, India

**Participants:** Mothers with children <2y (n=569)

**Main outcomes and analyses:** We measured HFI by using the Household Food Insecurity Access Scale and examined the changes in HFI during the pandemic using the Wilcoxon matched-pairs signed-rank tests. We then assessed child feeding practices and coping strategies by HFI status using multivariable regression models.

**Results**: HFI increased sharply from 21% in December 2019 to 80% in August 2020, with 62% households changing the status from food secure to insecure over this period. Children in newly or consistently food-insecure households were less likely to consume a diverse diet (adjusted odds ratio, AOR: 0.57, 95%CI: 0.34, 0.95 and AOR: 0.51, 95%CI: 0.23, 1.12, respectively) compared to those in food-secure households. Households with consistent food insecurity were more likely to engage in coping strategies such as reducing other essential non-food expenditures (AOR: 2.2, 95%CI: 1.09, 4.24), borrowing money to buy food (AOR: 4.3, 95%CI: 2.31, 7.95), or selling jewelry (AOR: 5.0, 95%CI: 1.74, 14.27) to obtain foods. Similar findings were observed for newly food-insecure households.

**Conclusions:** The COVID-19 pandemic and its lockdown measures posed a significant risk to HFI which in turn had implications for child feeding practices and coping strategies. Our findings




For peer review only - http://bmjopen.bmj.com/site/about/guidelines.xhtml

highlight the need for further investment in targeted social protection strategies and safety nets as part of multisectoral solutions to improve HFI during and after COVID-19.

**Keywords:** COVID-19, child feeding practices, coping strategies, household food insecurity, India, pandemic

For peer review only



For peer review only - http://bmjopen.bmj.com/site/about/guidelines.xhtml

**Strengths and limitations of this study**

- The longitudinal study design allowed measuring the impact of COVID-19 on HFI and its implications for child feeding practices and coping strategies in the context of low-and middle-income countries.
- The study demonstrated the feasibility of measuring HFI via digital data collection methods but indicated some challenges including low response rate and potential response bias.
- The sample of mothers with children <6 months constrained comparison of child feeding practices before and during COVID-19.
- A single point 24-hour dietary recall may be unrepresentative of child feeding patterns.
- The study was not able to assess whether the increase in level of HFI affected child growth.

For peer review only



For peer review only - http://bmjopen.bmj.com/site/about/guidelines.xhtml

## INTRODUCTION

The Coronavirus disease (COVID-19) has profound and wide-ranging public health impacts and poses a significant global threat to development. Beyond the direct impacts from the virus, the pandemic will likely have a range of indirect consequences on food insecurity, child malnutrition, morbidity and mortality through disruptions in health and nutrition services, food supply chains, and livelihoods.[1-4] Early estimates suggest that potential disruptions of health systems and decreased access to food could lead to 1,157,000 additional child deaths and 56,700 additional maternal deaths.[5] Further, disruptions caused by the pandemic may affect households in multiple other ways including employment and income loss, mobility constraints, and household stress. Experts have warned about the potential consequences of COVID-19, ruining decades of progress, making it unlikely for low and middle income countries to reach the sustainable development goal to "end hunger, achieve food security and improved nutrition and promote sustainable agriculture" by 2030.[6]

There have been growing concerns on the impact of COVID-19 on household food insecurity (HFI).[7 8] Disruptions caused by the pandemic have the potential to influence all "four pillars" of food security including availability, access, utilization, and stability.[9] The pandemic may influence HFI directly on the supply side by disrupting food systems (such as primary food production, stability of food production, processing, food reserve stockpiles, and marketing) as well as indirectly on the demand side due to impact of lockdowns on households' incomes, physical access to food, and economic access to food.[10 11]

The impact of COVID-19 on HFI and poor health outcomes is complex, multilevel, and bidirectional.[4] At the household and individual levels, food insecurity is hypothesized to be a risk factor for both short- and long-term health outcomes through key three pathways: household stress (due to worrying about health issues, job loss and strained finances, and disconnection from social

For peer review only

support systems), behavioral coping mechanisms (engaging in high-risk behavior, compromising health care activities for foods, poor mental health and inadequate child feeding and nurturing), and inflammatory pathways.[4] Expected negative consequences on food, nutrition, and health security of vulnerable groups including young children, pregnant, and lactating women may further exacerbate existing social and health inequities.[12]

Despite established frameworks and global understanding of the threat to HFI during the pandemic, empirical investigations are very limited to date. Available information on HFI was mainly collected during the pandemic [13-16] and very few studies have examined the dynamic changes of HFI over the COVID pandemic's evolution in low-and middle-income countries (LMICs) [17 18], particularly in the South Asian or Indian context. India is facing a double crisis- COVID-19 and food insecurity,[19] carrying the second highest burden of COVID-19 in the world with nearly 8 million total confirmed cases and 119,502 deaths as of 28th October 2020.[20] Yet only few studies are available on food security using data at the farmer and supply-side level,[21 22] and negligible evidence on the demand side. Very little is known about how women and children within households may be affected by HFI. Further, there is lack of empirical evidence on the changes in HFI during the pandemic. Addressing this knowledge gap is critical for action, specifically at this decisive time in India when the COVID-19 trajectory is still uncertain, and there is concern about potential spikes in the coming months. Our study seeks to address this gap in the current literature with the objectives to 1) assess the changes in HFI before and during the pandemic in Uttar Pradesh, India; and 2) examine the interlinkages between HFI with child feeding practices and coping strategies to deal with household economic hardships and obtain foods.



For peer review only - http://bmjopen.bmj.com/site/about/guidelines.xhtml

## METHODS

### Design

This study is a follow-up of a cluster-randomized trial (2017-2019) which aimed to assess the impact of strengthening delivery of maternal nutrition interventions, including micronutrient supplements and intensifying interpersonal counseling and community mobilization, implemented through government ANC services in Uttar Pradesh, India.[23] Details of the parent study have been described elsewhere.[24] Briefly, we conducted in-person repeated cross-sectional surveys of 1,800 recently delivered women as part of the cluster-randomized trial.[23] The end-line data collection was conducted in December 2019, prior to the onset of COVID-19 pandemic, providing an opportunity for a pre-and-post assessment of the effect of COVID-19 on food insecurity in this context.

### Data sources

The household survey was conducted with mothers of children <2 years old following the same study design and sampling frame as in the cluster-randomized trial. Of the 1,849 mothers surveyed at endline from the parent study in December 2019, 587 could be reached for a phone interview in August 2020, yielding a response rate of 32% **(Figure 1).** Reasons for not being able to conduct phone survey included unavailable phone number (n=388), phone unreachable or switched off (n=667), wrong number (n=136), refusal to participate (n=63) and child death (n=9). Reasons for losses to follow-up in the phone survey were similar between intervention and comparison areas (results not shown). The total sample of non-pregnant mothers (n=569) interviewed in both surveys were used for the analysis.

**<Insert Figure 1 here>**



For peer review only - http://bmjopen.bmj.com/site/about/guidelines.xhtml

**Variables**

Household food security was measured before (in-person) and during the pandemic (by phone) using the standard FANTA/USAID's Household Food Insecurity Access Scale.[25] A recent study in Mexico examined the internal validity of food insecurity scales administered through in-person vs. phone surveys and found phone surveys were a feasible strategy to measure food security during COVID-19.[18] Mothers were asked nine questions related to the household's experience of food insecurity in the 30 days preceding the survey. These questions capture 3 main domains of household food insecurity: anxiety and uncertainty about the household food supply (1 item), insufficient quality (3 items), and insufficient quantity and its physical consequences (5 items). We reported the percentage of households that experienced 1) any food insecurity occurrence among nine questions, 2) any of a specific domain, and 3) food insecurity condition categorized as food-secure and mild, moderately, or severely food-insecure.

Information on child feeding practices were assessed using the standard WHO indicators [26], on the basis of the maternal recall of all foods and liquids consumed by the child in different time periods of the previous 24 hours before the survey (**Supplemental Table 1**). All food items were categorized into the 7 food groups used in the WHO guideline:[26] 1) starchy staple foods, 2) legumes and nuts, 3) dairy products (milk, yogurt, and cheese), 4) flesh foods, 5) eggs, 6) vitamin A rich fruits and vegetables, and 7) other fruits and vegetables. Minimum dietary diversity was defined as children who consumed foods from 4 or more out of 7 food groups in the previous 24 hours. Data for complementary feeding practices were not available during the in-person survey in December 2019, because all mothers had children <6 months during that time.

Households were also asked about access to social protection, especially food supplementation they received for mothers and children from the government during the lockdown



For peer review only - http://bmjopen.bmj.com/site/about/guidelines.xhtml

period and during the 30 days prior to the survey, such as take-home rations (THR) and use of public distribution system (PDS). Finally, information on different coping strategies that the household had to engage in the past 30 days due to lack of food was collected, including spending savings, reducing essential non-food expenditure, borrowing money, or selling jewelry/gold.

Other potential factors associated with food security or child feeding practices were obtained for mothers (age, education level, and occupation), child (age and sex), and households (religion, scheduled caste/tribal - designated historically disadvantaged groups in India, number of children <5y, and household socio-economic status- SES). The SES index (collected in person survey) was constructed using a principal component analysis from multiple variables including household ownership of assets, livestock, and housing quality.[27]

**Data analysis**

We compared background characteristics of the analytic sample (mothers who completed both surveys, in-person survey before COVID and phone survey during COVID) and the non-analytic sample (those who completed in-person surveys only) using student t-test (for continuous variables) and chi-square test (for categorical variables). We used descriptive analysis to report HFI before and during the pandemic and child feeding practices. We examined changes in HFI before and during the pandemic using Wilcoxon matched-pairs signed-rank tests.

To examine differences in child feeding practices and coping strategies by food insecurity status, we created three categories of households: 1) food secure (households that were food secure before and during COVID-19 pandemic), 2) consistently food insecure (households that were food insecure before and during COVID-19); 3) newly food insecure (households that were food secure before COVID-19 but became food insecure during the pandemic). We then compared child feeding practices and coping strategies among the three categories using multivariable regression models,



For peer review only - http://bmjopen.bmj.com/site/about/guidelines.xhtml

For peer review only

adjusting for child age and sex, breastfeeding status, mother's age, religion, education, scheduled caste, number of children <5y in the household and household SES. We also examined uptake of social protection programs such as food supplementation and cash transfer as potential strategies to improve HFI. All statistical analyses were undertaken using Stata version 16. Statistical significance was defined as p-value < 0.05.

**Ethical approval**

Informed consent in the local language was obtained from mothers, frontline workers, and block managers prior to their participation in the study. The research protocol received ethical clearance from the Institutional Review Board at the International Food Policy Research Institute (IRB #00007490) and the Suraksha Independent Ethics Committee in India (IRB #2017-10-9094). Additional permissions for data collection were provided by the State Government of Uttar Pradesh.

**Patient and public involvement statement**

Patients or the public were not involved in the design, or conduct, or reporting, or dissemination plans of our research.

## RESULTS

**Characteristics of the study sample**

At the time of in-person survey in December 2019, all mothers had an infant between the ages of 0–5.9 months of age with an average age of 3 months (Table 1). On average, mothers were ~26 years and the majority of them (>90%) were housewives. Nearly all women were Hindu (92%) and nearly half of them belonged to a backward community (44-47%). Mothers in the final analytic sample had higher education (8.2 vs. 6.7 years of schooling, p<0.001) and lived in wealthier (27 vs. 17% in quintile 5, p<0.001) and more food secure households (79 vs. 75%, p=0.08) compared to



For peer review only - http://bmjopen.bmj.com/site/about/guidelines.xhtml

those in the non-analytic sample. Mothers belonging to intervention and control areas of the maternal nutrition intervention (from 2017 to 2019) were equally represented in the analytic sample. In the follow-up phone survey in August 2020- children were on average 11.6 months old (ranging between 8 and 14 months).

**Table 1: Background characteristics[1] of the study sample that participated in surveys before and during the COVID pandemic (December 2019 and August 2020)**

| | Analytic sample (both in person and phone surveys before and during the pandemic) (n= 569) | Non-analytic sample (only in person survey before the pandemic) (n= 1,280) | p |
|---|---|---|---|
| Age of respondent mother (years) | 25.5 (3.8) | 25.7 (4.0) | 0.47 |
| Education (years) | 8.2 (4.3) | 6.7 (4.6) | <0.001 |
| Never attended school | 14.1 | 24.8 | <0.001 |
| Primary school (grade1-5) | 13.9 | 16.3 | |
| Middle school (grade 6-9) | 24.3 | 24.7 | |
| High school (grade 10-12) | 30.1 | 23.3 | |
| Graduate and above | 17.8 | 10.9 | |
| Occupation as housewife | 91.7 | 93.0 | 0.35 |
| Child age, months | 3.0 (1.6) | 2.8 (1.6) | 0.041 |
| Child sex (male) | 49.0 | 49.5 | 0.84 |
| Number of children <5y | 1.6 (0.7) | 1.7 (0.7) | 0.60 |
| Religion as Hindu | 93.7 | 91.1 | 0.061 |
| Caste category | | | |
| Scheduled caste/tribe | 38.3 | 38.4 | 0.25 |
| Other Backward Class | 44.3 | 47.0 | |
| General/others | 17.4 | 14.5 | |
| Household socio-economic status | | | |
| Quintile 1 | 11.6 | 23.8 | <0.001 |
| Quintile 2 | 19.2 | 20.4 | |
| Quintile 3 | 18.1 | 20.9 | |
| Quintile 4 | 24.6 | 18.0 | |
| Quintile 5 | 26.5 | 17.0 | |
| Household food security status | | | |
| Food secure | 79.3 | 74.5 | 0.08 |
| Mildly food insecure | 5.6 | 5.9 | |
| Moderate food insecure | 5.1 | 5.3 | |


For peer review only - http://bmjopen.bmj.com/site/about/guidelines.xhtml

| | | |
|---|---|---|
| Severe food insecure | 10.0 | 14.3 |
| Maternal nutrition (2017-2019) | | |
| Intervention area | 282 | 640 |
| Comparison area | 287 | 640 |

[1]Background data presented in this table were from in -person survey in December 2019.

**Changes in food security status during the COVID pandemic**

Prior to the pandemic, 21% of households were identified as food insecure. Six months into the pandemic, the prevalence of any food insecurity increased from 21 to 80%, of which mildly, moderately, and severely food insecure households increased by 14 percentage points (pp), 25 pp and 20 pp, respectively (Figure 2A). Overall, 62% households changed from being food secure to food insecure during the pandemic. HFI experiences sharply increased for each domain. For example, the prevalence of anxiety and uncertainty about the household food supply, insufficient quality of food, and insufficient quantity of food consumed during the pandemic were 45, 78, and 42%, respectively, which was much higher than before the pandemic (12, 18, and 14%, respectively) (Figure 2B).

**<Insert Figure 2 here>**

**Child feeding practices during the COVID pandemic**

Child feeding practices are of major concern, with only 19% of children achieving minimum dietary diversity (≥ 4 food groups). An extremely low proportion of children were fed flesh foods (1%), eggs (1%) and vitamin-A rich fruits and vegetables (4%). One-third of the children consumed other fruits and vegetables and nearly two-thirds consumed legumes and nuts in the 24 hours prior to the survey (Figure 3).

**<Insert Figure 3 here>**

For peer review only

**Association between food insecurity status and child feeding practices during the COVID pandemic**

Children living in households that became food-insecure since the pandemic were less likely to consume a diversified diet (18 vs. 28%; adjusted odds ratio, AOR: 0.57, 95% CI: 0.34, 0.95) as well as legumes and nuts (57 vs. 69%; AOR: 0.61, 95% CI: 0.38, 0.97) compared to children living in consistently food secure households (Table 2).Child feeding practices were worse in the households that were food insecure at both times. Specifically, fewer children in consistently food-insecure households consumed a diverse diet (12.4 vs. 28%; AOR: 0.51, 95% CI: 0.23, 1.12) and other fruits and vegetables (21 vs. 40%; AOR: 0.50, 95% CI: 0.26, 0.97) compared to those in food secure households.


For peer review only - http://bmjopen.bmj.com/site/about/guidelines.xhtml

**Table 2: Association between child dietary diversity and household food insecurity status during the pandemic**

| | Currently food secure n=116 | Newly food insecure[1] n=354 | Consistent food insecurity n=99 | New food insecurity vs. food secure[2] | | | | Consistent food insecurity vs. food secure[2] | | | |
|---|---|---|---|---|---|---|---|---|---|---|---|
| | % | % | % | Crude OR (95%CI) | p | Adjusted OR (95%CI) | p | Crude OR (95%CI) | p | Adjusted OR (95%CI) | p |
| Grain | 79.3 | 80.8 | 78.8 | 1.1 (0.65,1.85) | 0.73 | 0.98 (0.57, 1.69) | 0.95 | 0.97 (0.50,1.87) | 0.93 | 0.87 (0.43, 1.77) | 0.64 |
| Legumes and nuts | 69.0 | 56.8 | 55.6 | 0.59 (0.38,0.93) | 0.02 | 0.61 (0.38, 0.97) | 0.04 | 0.56 (0.32,0.98) | 0.04 | 0.69 (0.38, 1.25) | 0.22 |
| Dairy | 74.1 | 76.3 | 79.8 | 1.12 (0.69,1.82) | 0.64 | 1.22 (0.74, 2.01) | 0.43 | 1.38 (0.72,2.62) | 0.33 | 1.72 (0.87, 3.41) | 0.12 |
| Flesh foods | 0.9 | 0.6 | 2.0 | 0.66 (0.06,7.36) | 0.74 | 0.63 (0.05, 7.47) | 0.72 | 2.37 (0.21,26.55) | 0.48 | 1.46 (0.09, 23.2) | 0.79 |
| Eggs | 0.9 | 1.1 | 1.0 | 1.33 (0.15,12.02) | 0.80 | 1.10 (0.11, 10.5) | 0.94 | 1.17 (0.07,19.01) | 0.91 | 0.87 (0.04, 17.0) | 0.93 |
| Vit A rich fruits and vegetables | 4.3 | 4.3 | 2.0 | 0.99 (0.35,2.79) | 0.99 | 0.77 (0.26, 2.26) | 0.64 | 0.46 (0.09,2.41) | 0.36 | 0.31 (0.05, 1.79) | 0.19 |
| Other fruits and vegetables | 39.7 | 33.5 | 21.2 | 0.77 (0.50,1.18) | 0.17 | 0.73 (0.46, 1.16) | 0.18 | 0.41 (0.22,0.75) | 0.004 | 0.50 (0.26, 0.97) | 0.042 |
| Minimum dietary diversity (≥ 4 food groups) | 28.1 | 17.9 | 12.4 | 0.56 (0.34,0.91) | 0.02 | 0.57 (0.34, 0.95) | 0.03 | 0.36 (0.17,0.75) | 0.006 | 0.51 (0.23, 1.12) | 0.09 |

[1]Currently food secure was defined as households who were food secure before and during COVID-19 pandemic and those who were food insecure at some point before but were no longer food insecure during the pandemic, consistent food insecurity was defined as both food insecure before and during COVID-19; newly food insecurity insecure was defined as food secure before COVID-19 but became food insecure during the pandemic. [2]Model controlled for child age, sex, breastfeeding status, mother's age, education, caste, religion, number of children<5y, and household SES.



For peer review only - http://bmjopen.bmj.com/site/about/guidelines.xhtml

For peer review only

**Challenges faced during the pandemic**

The key challenges faced by households in consuming food in the last 7 days preceding the survey included non-availability of funds to buy food (59%), non-availability of foods in market area (21%), increase in food prices (17%), and inability to travel or transport issues (21%). The pandemic-related challenges had resulted in unemployment/loss of income in 78.4% households (**Figure 4**).

**<Insert Figure 4 here>**

**Coping strategies and household food insecurity status during the COVID pandemic**

More than 60% of households disbursed their savings and reduced their expenses on health and non-food essentials to meet food and other requirements, irrespective of their food security status (Table 3). Households experiencing food insecurity were more likely to engage in coping strategies related to obtaining food including reducing their expenditure on non-food essentials (AOR: 1.7, 95% CI: 1.08, 2.78 and AOR: 2.2, 95% CI: 1.09, 4.24 for newly and consistently food-insecure households, respectively), borrowing money to buy food (AOR: 3.6, 95% CI: 2.19, 5.80 and AOR: 4.3, 95% CI: 2.31, 7.95, respectively), and selling jewelry (AOR: 3.0, 95% CI: 1.16, 7.92 and AOR: 5.0, 95% CI: 1.74, 14.27, respectively). Additionally, newly food-insecure households were ~2 times more likely to spend saving or sell households/assets/transport means.

For peer review only



For peer review only - http://bmjopen.bmj.com/site/about/guidelines.xhtml

**Table 3: Association between current coping strategies and household food insecurity status during the pandemic**

| | Currently food secure | Newly food insecure[1] | Consistent food insecurity | New food insecurity vs. food secure[2] | | | | Consistent food insecurity vs. food secure[2] | | | |
|---|---|---|---|---|---|---|---|---|---|---|---|
| | n=116 | n=354 | n=99 | | | | | | | | |
| | % | % | % | Crude OR (95%CI) | p | Adjusted OR (95%CI) | p | Crude OR (95%CI) | p | Adjusted OR (95%CI) | p |
| Spent savings | 83.6 | 91.0 | 89.9 | 1.97 (1.07,3.63) | 0.03 | 2.05 (1.09, 3.88) | 0.027 | 1.74 (0.77,3.95) | 0.18 | 1.73 (0.71, 4.18) | 0.23 |
| Reduced health expenditure | 64.7 | 72.0 | 74.7 | 1.41 (0.90,2.20) | 0.13 | 1.33 (0.84, 2.10) | 0.23 | 1.62 (0.90,2.92) | 0.11 | 1.49 (0.79, 2.80) | 0.22 |
| Reduced other essential non-food expenditures such as education and clothes | 66.4 | 77.4 | 81.8 | 1.73 (1.10,2.74) | 0.02 | 1.73 (1.08, 2.78) | 0.024 | 2.28 (1.20,4.32) | 0.01 | 2.15 (1.09, 4.24) | 0.027 |
| Borrowed money to buy food | 25.0 | 54.8 | 63.6 | 3.64 (2.27,5.82) | <0.001 | 3.57 (2.19, 5.80) | <0.001 | 5.25 (2.92,9.44) | <0.001 | 4.29 (2.31, 7.95) | <0.001 |
| Reduced expenses on agricultural, livestock or fisheries inputs | 23.3 | 33.3 | 35.4 | 1.65 (1.02,2.67) | 0.04 | 1.64 (0.99, 2.72) | 0.055 | 1.80 (0.99,3.27) | 0.05 | 1.78 (0.94, 3.38) | 0.078 |
| Selling jewelry/gold | 4.3 | 13.0 | 21.2 | 3.32 (1.28,8.56) | 0.01 | 3.03 (1.16, 7.92) | 0.024 | 5.98 (2.16,16.53) | 0.001 | 4.98 (1.74, 14.27) | 0.003 |
| Selling household goods or productive assets or means of transport | 19.0 | 29.4 | 27.3 | 1.78 (1.06,2.98) | 0.03 | 1.78 (1.03, 3.07) | 0.038 | 1.6 (0.84,3.04) | 0.15 | 1.64 (0.83, 3.26) | 0.16 |

[1]Currently food secure was defined as households who were food secure before and during COVID-19 pandemic and those who were food insecure at some point before but were no longer food insecure during the pandemic, consistent food insecurity was defined as both food insecure before and during COVID-19; newly food insecure was defined as food secure before COVID-19 but became food insecure during the pandemic. [2]Model controlled for mother's age, education, caste, religion, number of children<5y, and household SES.

For peer review only



For peer review only - http://bmjopen.bmj.com/site/about/guidelines.xhtml

**Social protection before and during the COVID pandemic**

The proportion of households where children received take-home rations (THR) from the ICDS program was similar before and during the pandemic and was slightly higher in food-insecure (~63%) compared to food-secure households (55-59%) (Figure 5).Coverage of PDS rations increased significantly during the pandemic for both food-insecure (61 to 71%) and food-secure households (from 49 to 72%); the increase was smaller among beneficiaries from consistently food-insecure compared to those in food-secure households (9.3 pp vs. 23 pp).

**<Insert Figure 5 here>**

## DISCUSSION

In response to global concerns on the impact of COVID-19 on maternal and child food and nutrition insecurity, our study provides unique evidence of changes in HFI before and during the pandemic and its linkages with child feeding practices as well as coping strategies to obtain foods among food secure and insecure households. We found that HFI increased substantially during the pandemic (60 pp), with a large portion related to insufficient quality (78%) and lower levels related to insufficient quantity (42%). Children living in food-insecure households were less likely to consume a diversified diet, mainly due to less consumption of legumes and nuts, fruits, and vegetables. In order to overcome the challenges during the pandemic, households were compelled to engage in several coping strategies related to spending existing savings, reducing household expenditures, selling assets, or borrowing money.

Our findings were consistent with the global literature on the increase in HFI during the pandemic.[28-32] However, most previous studies mainly obtained information during the pandemic and did not have data prior to the onset of the pandemic. A rapid assessment conducted in LMICs including Kenya, Nigeria, Mozambique, and Rwanda showed that 79-87% of



For peer review only - http://bmjopen.bmj.com/site/about/guidelines.xhtml

respondents were worried about lack of sufficient food during COVID-19.[13] Similarly, nearly 90% of households in rural and urban Bangladesh experienced different levels of food insecurity and engaged in financial or food compromised coping strategies.[15] The prevalence of moderate to severe HFI during the COVID-19 lockdown was lower in Peru, affecting 23% of households, with predictors being low income pre-pandemic, income reduction, or running out of savings during the pandemic.[14] Among the few studies with information before and during COVID-19 time, two were from the US, one found 32% increase in HFI since COVID-19 [16] while the other found an increase of 20%.[28] Only two other studies provided estimates of HFI before and during COVID where one found an increase of 14 pp (from 61.1 to 75.1%) in any HFI in Mexico[18] and the other observed an increase of 43.4 pp (from 8.3 to 51.7%) in moderate and severe HFI in Bangladesh.[17] Our study showed much higher magnitude of increase in HFI (~60 pp) compared to other studies, which is a worrisome finding given the high pre-existing levels of food insecurity in India. We also found that HFI was predominantly due to insufficient food quality concerns which was aligned with a previous study which showed increased consumption of high-calorie snack foods and sweets,[28 30] or cheaper highly processed foods.[4]

Our findings indicate challenges to several food security dimensions, including livelihood and income loss, economic and physical access, availability, and utilization. A study on livelihood and dietary effects of COVID-19 with vegetable producers in four states of India reported negative impacts on production, sales, prices, and incomes among majority of farmers,[21] Farm households also reported disruptions to their diets with reduced ability to access nutrient-dense foods, particularly fruit and animal source foods.[21] Another study in Maharashtra, India found disruptions in the urban-rural food supply chain due to the closure of wholesale markets with uncertainties in food supply, declines in market availability, and increase in food prices.[22]



For peer review only - http://bmjopen.bmj.com/site/about/guidelines.xhtml

These findings are complementary to our study and the supply-side insights possibly explain some of the trends we observe in food security, child feeding, and coping strategies.

To our knowledge, infant and child feeding practices during the pandemic have not been explored in the literature. Our findings showed that the diets of children were suboptimal, with only 19% achieving minimum dietary diversity – a similar result compared to a previous study in Uttar Pradesh, India before COVID pandemic (17%).[33] We also found that children living in food-insecure households had much poorer diets than those in food-secure household, but the proportion of children consuming flesh foods, eggs, and vitamin A fruits and vegetables is very low, irrespective of food security status. During the COVID-19 pandemic, child feeding practices have been reported to change, particularly among food-insecure households, due to higher levels of stress, fewer resources, and less access to food and affordability, leading to restrict the quantity and quality of food their children eat and more parents' controlling feeding behaviors.[28] Other studies also showed that mothers of the children in food-insecure households often prioritized shelf-stable foods to deal with food supply disruptions and social-distancing policies, and have a tendency to rely on energy-dense foods for a longer period of time.[4]

We found that all households in our study engaged in some coping strategies to obtain food regardless of HFI status, but food-insecure households were more likely to engage in several such practices. Our findings are consistent with literature stating that the main strategies food-insecure households generally rely on to maintain access to food include shifting within their own spending patterns to prioritize food (reducing expenses on health, other non-food expenditures, or agricultural, livestock or fisheries inputs), relying on social network, or access to government nutrition programs.[4 16] However, all these strategies can easily be impacted when COVID pandemic severely affects the entire household budget, or social-distancing policies


For peer review only - http://bmjopen.bmj.com/site/about/guidelines.xhtml

could affect network access. The coping strategies households have adopted to obtain food during COVID-19 will run out and will not suffice for preventing HFI from getting worse if the pandemic crisis continues.

Social protection strategies are an important intervention to address the rising levels of HFI in the context of COVID-19, particularly for low-income countries.[34] A global review of evidence by the World Bank found that India increased coverage of cash transfers from ~2% before the pandemic to about 15% during COVID-19.[35] The Indian government also initiated home-delivery of take-home rations for pregnant and lactating women and children and provided one-month free supply of wheat and rice to the poorest ration card holders through the public distribution system.[36] Our findings on the increased access to PDS during COVID-19 align with previous conclusions about the important role of the program as an essential component of the Government's response to food insecurity.[37] Despite these measures, food supplementation was received among just over half of households and the increase in access to PDS was smaller among beneficiaries that are consistently food insecure compared to the food secure. These results highlight an important opportunity to strengthen the Government's response to reduce food insecurity during and after COVID-19 in the short term by improving efficiency of existing social protection strategies and targeting to the most vulnerable populations.[37 38] A recent costing study conducted in Mexico found it would cost less than 0.06% of the Gross Domestic Product to effectively safeguard families with young children through a cash transfer and basic services subsidy.[39] Other strategies which may be considered include outlining specific recommendations to ensure food security for poor and vulnerable populations as done for other developing countries in Africa[40] and include special initiatives for migrant populations.[41] Certain agricultural reforms may also be considered[42] such as home gardening,[43] diversification of production, and



For peer review only - http://bmjopen.bmj.com/site/about/guidelines.xhtml

strong local market chains[44] to alleviate HFI, improve diets, and reduce reliance on coping strategies due to food insecurity.

Our study followed the cohort of mothers before the pandemic and 6 months after the onset of COVID-19, thus offering a unique and timely contribution to the literature on the magnitude and nature of increase in HFI before and during the pandemic, and its implications for child feeding practices and coping strategies in the context of LMICs with prevailing high HFI. Given the restrictions on movement and contacting people, we were able to mobilize the phone survey to reach mothers and use the same instrument to measure food security over time. Our experience demonstrated the feasibility of gathering information on HFI via digital data collection methods but indicated potential challenges and bias in the background characteristics of respondents interviewed through in-person vs. phone surveys. Mothers who responded to phone survey had slightly higher education and SES background compared to non-responders, indicating that we may not be able to reach some of the poorest or most vulnerable households through phone surveys. We also experienced similar challenges as other phone surveys[45] including low response rate, several calling schedules during the survey, and potential unknown response bias or residual confounding factors. Since all mothers in our study had children <6 months in December 2019, we were unable to obtain information on complementary feeding to compare child feeding practices before and during COVID time. Child feeding was assessed by a single point 24-hour dietary recall which may be unrepresentative of overall dietary exposure. Finally, we were not able to assess whether the increase in level of HFI affected child growth which should be considered in future research.



For peer review only - http://bmjopen.bmj.com/site/about/guidelines.xhtml

## CONCLUSION

The COVID-19 pandemic and lockdown measures arising from the pandemic had a significant negative impact on HFI in this context, which in turn had implications for child feeding practices and reliance on coping strategies to obtain foods. Our study highlighted the opportunity to reduce HFI in the short-term with existing resources by improving the targeting of social protection benefits to effectively reach the food insecure and make quality diets accessible. Given the great concerns about the expected increase in HFI as the pandemic continues, strengthened multisectoral responses are needed to ensure effective re-establishment of health and nutrition services, food supply chains, and restoration of livelihoods to improve household food security during and after the pandemic. Policies response to the pandemic also require coordination across different governance systems to guide threat against HFI in future pandemics because the most important impact on food security is related to a serious slowdown in economic activity and disrupted supply chains caused by strict lockdown measures, not the pandemic itself.

For peer review only



For peer review only - http://bmjopen.bmj.com/site/about/guidelines.xhtml

## FIGURE LEGENDS

**Figure 1.** Participant flow

**Figure 2.** Food insecurity experienced by mothers and their household members in the previous 30 days before and during the COVID pandemic

**Figure 3:** Child feeding practices during the COVID pandemic

**Figure 4:** The key challenges faced by households during the COVID pandemic

**Figure 5:** Household receipt of social protection benefits before and during the pandemic, by household food insecurity status

For peer review only



For peer review only - http://bmjopen.bmj.com/site/about/guidelines.xhtml

## AUTHOR CONTRIBUTIONS

PHN: conceived paper, analysis, drafted manuscript, consolidated comments from all co-authors, revised and finalized paper.

SK: Field work coordination, literature review, drafted some parts of the manuscript, revised, and finalized paper.

AP: Field work coordination, data analyses, drafted some parts of the manuscript, reviewed manuscript.

LMT: data analyses, visualization for data presentation, reviewed the manuscript.

SG, PKS, VDS, JE: data interpretation and its implications, reviewed and edited the manuscript.

RA and PM reviewed the statistical analyses, supported data interpretation, reviewed, and edited the manuscript.

All authors read and approved the final submitted manuscript.

## SOURCE OF FUNDING

Bill & Melinda Gates Foundation through POSHAN, led by International Food Policy Research Institute. Grant number: OPP50838.

## CONFLICT OF INTEREST STATEMENT

Authors declare no conflicts of interest.

## DATA AVAILABILITY STATEMENT

All data relevant to the study are included in the article or uploaded as supplementary information.



For peer review only - http://bmjopen.bmj.com/site/about/guidelines.xhtml

## REFERENCES


1. Tesfaye A, Habte Y, Minten B. COVID-19 is shifting consumption and disrupting dairy value chains in Ethiopia. IFPRI book chapters, in: COVID-19 and global food security, chapter 9, pages 42-45, International Food Policy Research Institute (IFPRI). 2020
2. Hirvonen K, De Brauw A, Abate G. Food Consumption and Food Security during the COVID-19 Pandemic in Addis Ababa. IFPRI Discussion Paper 01964. 2020
3. Headey D, Heidkamp R, Osendarp S, et al. Impacts of COVID-19 on childhood malnutrition and nutrition-related mortality. *Lancet* 2020;396(10250):519-21. doi: 10.1016/S0140-6736(20)31647-0 [published Online First: 2020/07/31]
4. Leddy AM, Weiser SD, Palar K, et al. A conceptual model for understanding the rapid COVID-19-related increase in food insecurity and its impact on health and healthcare. *Am J Clin Nutr* 2020 doi: 10.1093/ajcn/nqaa226 [published Online First: 2020/08/09]
5. Roberton T, Carter ED, Chou VB, et al. Early estimates of the indirect effects of the COVID-19 pandemic on maternal and child mortality in low-income and middle-income countries: a modelling study. *Lancet Glob Health* 2020;8(7):e901-e08. doi: 10.1016/S2214-109X(20)30229-1 [published Online First: 2020/05/15]
6. United Nations. Resolution adopted by the General Assembly on 25 September 2015, Transforming our world: the 2030 Agenda for Sustainable Development (A/RES/70/1) 2015
7. Paslakis G, Dimitropoulos G, Katzman DK. A call to action to address COVID-19-induced global food insecurity to prevent hunger, malnutrition, and eating pathology. *Nutr Rev* 2020 doi: 10.1093/nutrit/nuaa069 [published Online First: 2020/07/12]
8. Cable J, Jaykus LA, Hoelzer K, et al. The impact of COVID-19 on food systems, safety, and security-a symposium report. *Ann N Y Acad Sci* 2020 doi: 10.1111/nyas.14482 [published Online First: 2020/08/30]
9. Laborde D, Martin W, Swinnen J, et al. COVID-19 risks to global food security. *Science* 2020;369(6503):500-02. doi: 10.1126/science.abc4765 [published Online First: 2020/08/01]
10. Devereux S, Bene C, Hoddinott J. Conceptualising COVID-19's impacts on household food security. *Food Secur* 2020:1-4. doi: 10.1007/s12571-020-01085-0 [published Online First: 2020/08/25]
11. Savary S, Akter S, Almekinders C, et al. Mapping disruption and resilience mechanisms in food systems. *Food Secur* 2020:1-23. doi: 10.1007/s12571-020-01093-0 [published Online First: 2020/08/25]
12. Perez-Escamilla R, Cunningham K, Moran VH. COVID-19 and maternal and child food and nutrition insecurity: a complex syndemic. *Matern Child Nutr* 2020;16(3):e13036. doi: 10.1111/mcn.13036 [published Online First: 2020/05/28]
13. GAIN. Impact of COVID-19 on Food Systems: A Situation Report, Edition 3. May 13, 2020. 2020
14. Cañari-Casaño JL, Elorreaga OA, Cochachin-Henostroza O, et al. Social predictors of food insecurity during the stay-at-home order due to the COVID-19 pandemic in Peru. Results from a cross-sectional web-based survey. *medRxiv 2021 Feb 8;2021020621251221 doi: 101101/2021020621251221 Preprint* 2021
15. Das S, Rasul MG, Hossain MS, et al. Acute food insecurity and short-term coping strategies of urban and rural households of Bangladesh during the lockdown period of COVID-19




For peer review only - http://bmjopen.bmj.com/site/about/guidelines.xhtml

pandemic of 2020: report of a cross-sectional survey. *BMJ Open* 2020;10(12):e043365. doi: 10.1136/bmjopen-2020-043365 [published Online First: 2020/12/15]

16. Niles MT, Bertmann F, Belarmino EH, et al. The Early Food Insecurity Impacts of COVID-19. *Nutrients* 2020;12(7) doi: 10.3390/nu12072096 [published Online First: 2020/07/19]
17. Hamadani JD, Hasan MI, Baldi AJ, et al. Immediate impact of stay-at-home orders to control COVID-19 transmission on socioeconomic conditions, food insecurity, mental health, and intimate partner violence in Bangladeshi women and their families: an interrupted time series. *Lancet Glob Health* 2020;8(11):e1380-e89. doi: 10.1016/S2214-109X(20)30366-1 [published Online First: 2020/08/29]
18. Gaitan-Rossi P, Vilar-Compte M, Teruel G, et al. Food insecurity measurement and prevalence estimates during the COVID-19 pandemic in a repeated cross-sectional survey in Mexico. *Public Health Nutr* 2021;24(3):412-21. doi: 10.1017/S1368980020004000 [published Online First: 2020/10/15]
19. Mishra K, Rampal J. The COVID-19 pandemic and food insecurity: A viewpoint on India. *World Development* *https://doiorg/101016/jworlddev2020105068* 2020;135:1-3.
20. Ministry of Health and Family Welfare. COVID-19 INDIA as on : 14 October 2020, 08:00 IST (GMT+5:30) New Delhi, India: Government of India; 2020 [Available from: https://www.mohfw.gov.in/index1.php?lang=1level=1sublinkid=6471lid=4270 accessed 15th October 2020.
21. Harris J, Depenbusch L, Pal AA, et al. Food system disruption: initial livelihood and dietary effects of COVID-19 on vegetable producers in India. *Food Secur* 2020:1-11. doi: 10.1007/s12571-020-01064-5
22. Sukhwani V, Deshkar S, Shaw R. COVID-19 Lockdown, Food Systems and Urban-Rural Partnership: Case of Nagpur, India. *Int J Environ Res Public Health* 2020;17(16) doi: 10.3390/ijerph17165710 [published Online First: 2020/08/14]
23. Nguyen P, Kachwaha S, Tran LM, et al. Strengthening Nutrition Interventions in Antenatal Care Services Had Modest Impacts on Diet Diversity, Micronutrient Intake, and Breastfeeding in Uttar Pradesh, India. *Current Developments in Nutrition,* 2020;4(Supplement_2):1050, https://doi.org/10.93/cdn/nzaa054_122.
24. Nguyen PH, Kachwaha S, Tran LM, et al. Integrating Maternal Nutrition Interventions in Existing Reproductive, Maternal, Newborn and Child Health Services in Uttar Pradesh, India. Alive & Thrive Endline Survey Report. Washington, DC: Alive & Thrive. 2020
25. Coates J, Swindale A, Bilinsky P. Household Food Insecurity Access Scale (HFIAS) for Measurement of Household Food Access: Indicator Guide (v. 3). Washington, D.C.: Food and Nutrition Technical Assistance Project, Academy for Educational Development. 2007
26. WHO. Indicators for assessing infant and young child feeding practices. Part 2: Measurements. http://whqlibdoc.who.int/publications/2010/9789241599290_eng.pdf. World Health Organization: Geneva, 2008.
27. Filmer D, Pritchett LH. Estimating wealth effects without expenditure data--or tears: an application to educational enrollments in states of India. *Demography* 2001;38(1):115-32. [published Online First: 2001/03/03]
28. Adams EL, Caccavale LJ, Smith D, et al. Food insecurity, the home food environment, and parent feeding practices in the era of COVID-19. *Obesity (Silver Spring)* 2020 doi: 10.1002/oby.22996 [published Online First: 2020/08/08]



For peer review only - http://bmjopen.bmj.com/site/about/guidelines.xhtml

29. Bene C. Resilience of local food systems and links to food security - A review of some important concepts in the context of COVID-19 and other shocks. *Food Secur* 2020:1-18. doi: 10.1007/s12571-020-01076-1 [published Online First: 2020/08/25]
30. Carroll N, Sadowski A, Laila A, et al. The Impact of COVID-19 on Health Behavior, Stress, Financial and Food Security among Middle to High Income Canadian Families with Young Children. *Nutrients* 2020;12(8) doi: 10.3390/nu12082352 [published Online First: 2020/08/14]
31. Kent K, Murray S, Penrose B, et al. Prevalence and Socio-Demographic Predictors of Food Insecurity in Australia during the COVID-19 Pandemic. *Nutrients* 2020;12(9) doi: 10.3390/nu12092682 [published Online First: 2020/09/06]
32. Owens MR, Brito-Silva F, Kirkland T, et al. Prevalence and Social Determinants of Food Insecurity among College Students during the COVID-19 Pandemic. *Nutrients* 2020;12(9) doi: 10.3390/nu12092515 [published Online First: 2020/08/23]
33. MoHFW. Ministry of Health and Family Welfare (MoHFW), Government of India, UNICEF and Population Council. Comprehensive National Nutrition Survey (CNNS) National Report. New Delhi, India, 2019.
34. Pereira M, Oliveira AM. Poverty and food insecurity can increase as threats of COVID-19 spreads. *Public Health Nutr* 2020:1-10. doi: 10.1017/S1368980020003493 [published Online First: 2020/09/09]
35. Gentilini U, Almenfi M, Orton I, et al. Social Protection and Jobs Responses to COVID-19 : A Real-Time Review of Country Measures. Washington DC: World Bank, 2020.
36. International Food Policy Research Institute. POSHAN COVID-19 Resources: Online archive for COVID-19 related resources on nutrition, early childhood development and food security New Delhi, India: POSHAN; 2020 [Available from: https://poshancovid19.in/Resources.html accessed 15th October 2020.
37. George NA, McKay FH. The Public Distribution System and Food Security in India. *Int J Environ Res Public Health* 2019;16(17) doi: 10.3390/ijerph16173221 [published Online First: 2019/09/06]
38. FAO, IFAD, UNICEF, et al. The State of Food Security and Nutrition in the World 2020. Transforming food systems for affordable healthy diets. Rome, FAO. DOI: https://doi.org/10.4060/ca9692en. 2020
39. Vilar-Compte M, Perez V, Teruel G, et al. Costing of actions to safeguard vulnerable Mexican households with young children from the consequences of COVID-19 social distancing measures. *Int J Equity Health* 2020;19(1):70. doi: 10.1186/s12939-020-01187-3 [published Online First: 2020/05/21]
40. Lawson-Lartego L, Cohen MJ. 10 recommendations for African governments to ensure food security for poor and vulnerable populations during COVID-19. *Food Secur* 2020;12(4):899-902. doi: 10.1007/s12571-020-01062-7 [published Online First: 2020/08/26]
41. Smith MD, Wesselbaum D. COVID-19, Food Insecurity, and Migration. *J Nutr* 2020 doi: 10.1093/jn/nxaa270 [published Online First: 2020/08/26]
42. Kumar A, Padhee AK, Kumar S. How Indian agriculture should change after COVID-19. *Food Secur* 2020:1-4. doi: 10.1007/s12571-020-01063-6 [published Online First: 2020/08/25]



For peer review only - http://bmjopen.bmj.com/site/about/guidelines.xhtml

43. Lal R. Home gardening and urban agriculture for advancing food and nutritional security in response to the COVID-19 pandemic. *Food Secur* 2020:1-6. doi: 10.1007/s12571-020-01058-3 [published Online First: 2020/08/25]
44. Heck S, Campos H, Barker I, et al. Resilient agri-food systems for nutrition amidst COVID-19: evidence and lessons from food-based approaches to overcome micronutrient deficiency and rebuild livelihoods after crises. *Food Security* *https://doiorg/101007/s12571-020-01067-2* 2020
45. Kempf AM, Remington PL. New challenges for telephone survey research in the twenty-first century. *Annu Rev Public Health* 2007;28:113-26. doi: 10.1146/annurev.publhealth.28.021406.144059 [published Online First: 2006/11/11]

For peer review only



For peer review only - http://bmjopen.bmj.com/site/about/guidelines.xhtml

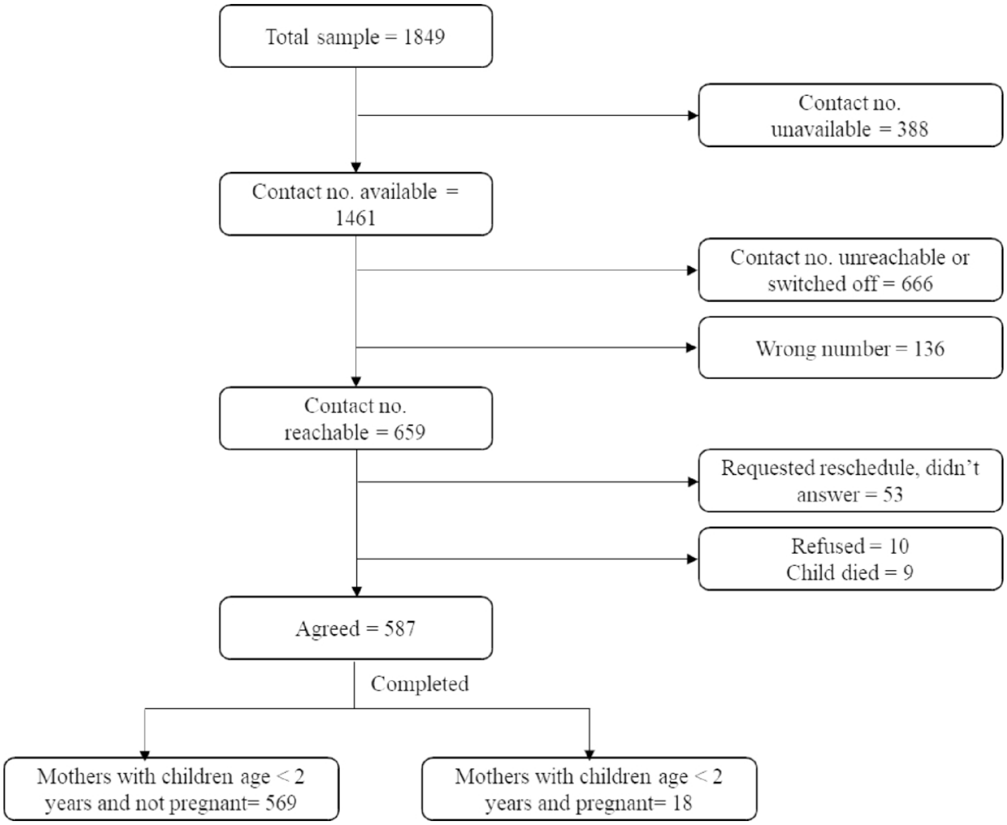


Figure 1. Participant flow

172x140mm (300 x 300 DPI)

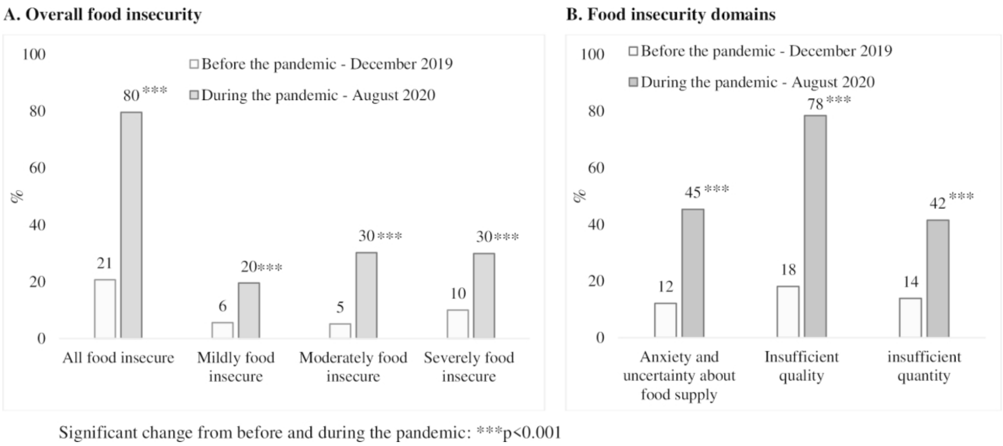


Figure 2. Food insecurity experienced by mothers and their household members in the previous 30 days before and during the COVID pandemic

190x82mm (300 x 300 DPI)

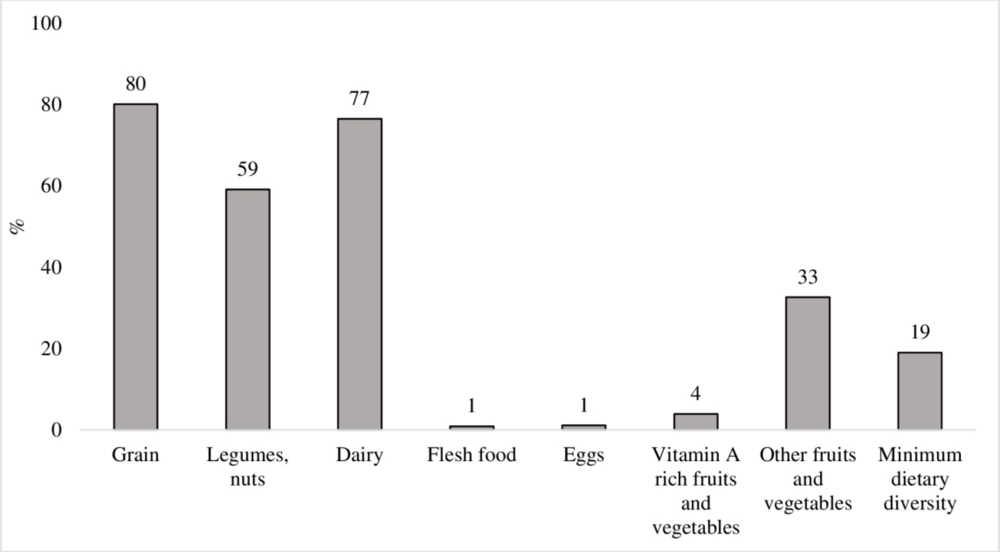


Figure 3: Child feeding practices during the COVID pandemic

168x92mm (300 x 300 DPI)

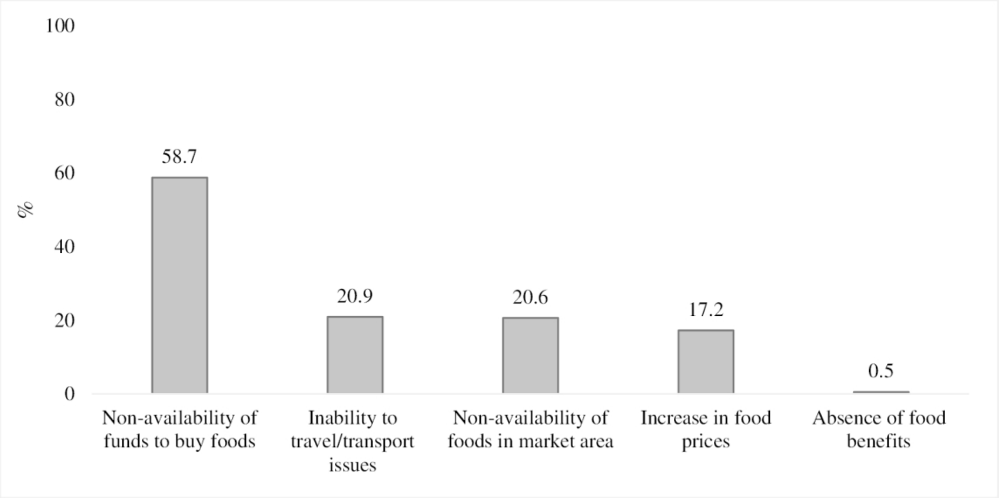


Figure 4: The key challenges faced by households during the COVID pandemic

156x77mm (300 x 300 DPI)

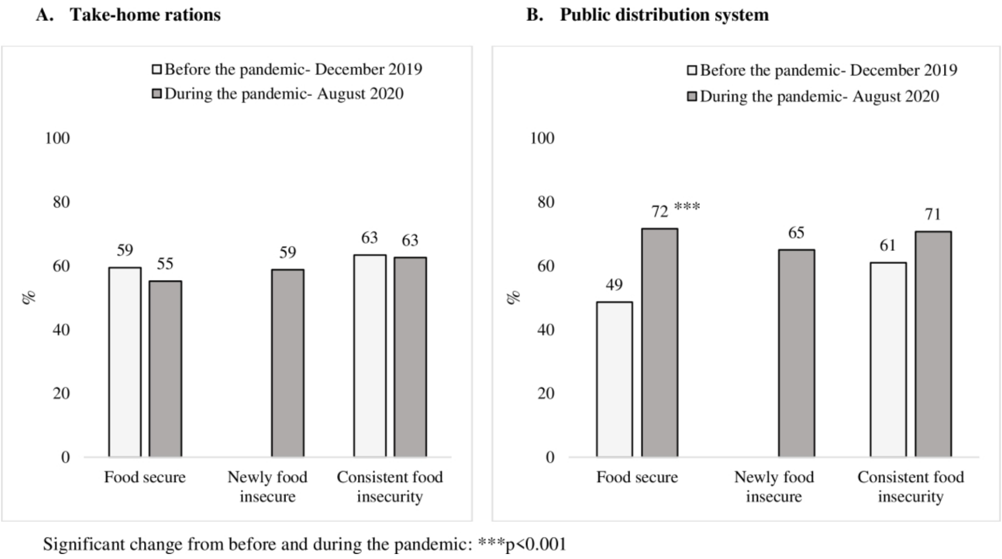


Figure 5: Household receipt of social protection benefits before and during the pandemic, by household food insecurity status

187x102mm (300 x 300 DPI)

**Supplemental Table 1: Food items from the maternal recall in the 24 hours prior to the survey**

| No | Food items |
|---|---|
| 1. | Porridge, bread, rice, noodles, or other foods made from grains |
| 2. | Pumpkin, carrots, squash, or sweet potatoes that are yellow or orange inside |
| 3. | White potatoes, white yams, manioc, cassava, or any other foods made from roots |
| 4. | Any dark green leafy vegetables |
| 5. | Ripe mangoes, ripe papayas, or other local vitamin A-rich fruits |
| 6. | Any other fruits or vegetables |
| 7. | Liver, kidney, heart, or other organ meats |
| 8. | Any meat, such as beef, pork, lamb, goat, chicken, or duck |
| 9. | Eggs |
| 10. | Fresh or dried fish, shellfish, or seafood |
| 11. | Any foods made from beans, peas, lentils, nuts, or seeds |
| 12. | Cheese, yogurt, or other milk products |
| 13. | Any oil, fats, or butter, or foods made with any of these |
| 14. | Any sugary foods such as chocolates, sweets, candies, pastries, cakes, or biscuits |
| 15. | Condiments for flavor, such as chilies, spices, herbs, or fish powder |
| 16. | Grubs, snails, or insects |
| 17. | Foods made with red palm oil, red palm nut, or red palm nut pulp sauce |
| 18. | Baby formula |

For peer review only

**STROBE Statement—Checklist of items that should be included in reports of cross-sectional studies**

| | Item No | Recommendation | Page number from manuscript |
|---|---|---|---|
| **Title and abstract** | 1 | (*a*) Indicate the study's design with a commonly used term in the title or the abstract | 2 |
| | | (*b*) Provide in the abstract an informative and balanced summary of what was done and what was found | 2-3 |
| **Introduction** | | | |
| Background/rationale | 2 | Explain the scientific background and rationale for the investigation being reported | 5-6 |
| Objectives | 3 | State specific objectives, including any prespecified hypotheses | 6 |
| **Methods** | | | |
| Study design | 4 | Present key elements of study design early in the paper | 7 |
| Setting | 5 | Describe the setting, locations, and relevant dates, including periods of recruitment, exposure, follow-up, and data collection | 7-9 |
| Participants | 6 | (*a*) Give the eligibility criteria, and the sources and methods of selection of participants | 7-9 |
| Variables | 7 | Clearly define all outcomes, exposures, predictors, potential confounders, and effect modifiers. Give diagnostic criteria, if applicable | 8-9 |
| Data sources/ measurement | 8* | For each variable of interest, give sources of data and details of methods of assessment (measurement). Describe comparability of assessment methods if there is more than one group | 8-9 |
| Bias | 9 | Describe any efforts to address potential sources of bias | NA |
| Study size | 10 | Explain how the study size was arrived at | 7 |
| Quantitative variables | 11 | Explain how quantitative variables were handled in the analyses. If applicable, describe which groupings were chosen and why | 9-10 |
| Statistical methods | 12 | (*a*) Describe all statistical methods, including those used to control for confounding | 9-10 |
| | | (*b*) Describe any methods used to examine subgroups and interactions | 9-10 |
| | | (*c*) Explain how missing data were addressed | NA |
| | | (*d*) If applicable, describe analytical methods taking account of sampling strategy | NA |
| | | (*e*) Describe any sensitivity analyses | NA |
| **Results** | | | |
| Participants | 13* | (a) Report numbers of individuals at each stage of study—eg numbers potentially eligible, examined for eligibility, confirmed eligible, included in the study, completing follow-up, and analysed | 10 |
| | | (b) Give reasons for non-participation at each stage | 7 |
| | | (c) Consider use of a flow diagram | Figure 1 |
| Descriptive data | 14* | (a) Give characteristics of study participants (eg demographic, clinical, social) and information on exposures and potential | 10-11 |

For peer review only


For peer review only - http://bmjopen.bmj.com/site/about/guidelines.xhtml

| | | | |
|---|---|---|---|
| | | confounders | |
| | | (b) Indicate number of participants with missing data for each variable of interest | 10-11 |
| Outcome data | 15* | Report numbers of outcome events or summary measures | 12-15 |
| Main results | 16 | (*a*) Give unadjusted estimates and, if applicable, confounder-adjusted estimates and their precision (eg, 95% confidence interval). Make clear which confounders were adjusted for and why they were included | 12-15 |
| | | (*b*) Report category boundaries when continuous variables were categorized | NA |
| | | (*c*) If relevant, consider translating estimates of relative risk into absolute risk for a meaningful time period | NA |
| Other analyses | 17 | Report other analyses done—eg analyses of subgroups and interactions, and sensitivity analyses | NA |
| **Discussion** | | | |
| Key results | 18 | Summarise key results with reference to study objectives | 16 |
| Limitations | 19 | Discuss limitations of the study, taking into account sources of potential bias or imprecision. Discuss both direction and magnitude of any potential bias | 19 |
| Interpretation | 20 | Give a cautious overall interpretation of results considering objectives, limitations, multiplicity of analyses, results from similar studies, and other relevant evidence | 16-19 |
| Generalisability | 21 | Discuss the generalisability (external validity) of the study results | 19-20 |
| **Other information** | | | |
| Funding | 22 | Give the source of funding and the role of the funders for the present study and, if applicable, for the original study on which the present article is based | 22 |

*Give information separately for exposed and unexposed groups.

**Note:** An Explanation and Elaboration article discusses each checklist item and gives methodological background and published examples of transparent reporting. The STROBE checklist is best used in conjunction with this article (freely available on the Web sites of PLoS Medicine at http://www.plosmedicine.org/, Annals of Internal Medicine at http://www.annals.org/, and Epidemiology at http://www.epidem.com/). Information on the STROBE Initiative is available at www.strobe-statement.org.

For peer review only



For peer review only - http://bmjopen.bmj.com/site/about/guidelines.xhtml
